# LINCRNA00273 promotes cancer metastasis and its G-Quadruplex promoter can serve as a novel target to inhibit cancer invasiveness

**DOI:** 10.18632/oncotarget.22622

**Published:** 2017-11-17

**Authors:** Samarjit Jana, Jagannath Jana, Kartick Patra, Soma Mondal, Jyotsna Bhat, Arnab Sarkar, Pallabi Sengupta, Anindya Biswas, Meghomukta Mukherjee, Satya Prakash Tripathi, Rahul Gangwal, Joyita Hazra, Abhay T. Sangamwar, Gopeswar Mukherjee, Shamee Bhattacharjee, Deba Prasad Mandal, Subhrangsu Chatterjee

**Affiliations:** ^1^ Department of Biophysics, Bose Institute, P-1/12 CIT Scheme VIIM, Kankurgachi, Kolkata 700054, India; ^2^ Department of Zoology, West Bengal State University, Malikapur, Kolkata 700126, India; ^3^ Department of Biochemistry, Bose Institute, P-1/12 CIT Scheme VIIM, Kankurgachi, Kolkata 700054, India; ^4^ Barasat Cancer Research and Welfare Centre, Barasat, Kolkata 700124, India; ^5^ Department of Pharmacoinformatics, National Institute of Pharmaceutical Education and Research (NIPER), Sector-67, S. A. S. Nagar, Punjab 160062, India; ^6^ Department of Molecular Medicine, Bose Institute, P-1/12 CIT Scheme VIIM, Kankurgachi Kolkata 700054, India; ^7^ Department of Pharmaceutics, National Institute of Pharmaceutical Education and Research (NIPER), Sector-67, S. A. S. Nagar, Punjab 160062, India

**Keywords:** long non coding intergenic RNA, cancer metastasis, G-quadruplex, epithelial to mesenchymal transition

## Abstract

Discovery of anti-metastatic drugs is of immense clinical significance as metastasis is responsible for 90% of all cancer deaths. Here we report the inhibitory effect of a bis schiff base (M2) on cancer cell migration and invasion *in vitro* and *in vivo*. M2 has shown good solubility and permeability across the intestinal cell wall and hence can be classified as BCS (Biopharmaceutical classification system) class I. Microarray studies identified a long non coding intergenic RNA, LINC00273 as a novel molecular target of M2. We report that LINC00273 harbors a unique (4n-1) parallel G-Quadruplex structure in its promoter as validated by DMS footprint. M2 is proposed to stabilize this G-quadruplex structure resulting in the down-regulation of LINC00273 expression. Dual Luciferase reporter assay also suggests inhibition of LINC00273 promoter activity by M2. Involvement of this linc in metastasis is proven by siRNA and shRNA mediated knock down of LINC00273 *in vitro* and *in vivo* in nude mice which significantly decelerates cancer cell migration and invasion and also makes the cells unresponsive to TGF-β's pro-metastatic effects. Furthermore, the real time expression of LINC00273 in thirty seven human clinical samples is found to be positively correlated with the histopathological staging of metastasis.

## INTRODUCTION

Despite significant advances in cancer therapy, cancer still continues to be the leading cause of mortality worldwide. According to the latest GLOBOCAN report produced by the International Agency for Research on Cancer (IARC), about 14.1 million new cancer cases and 8.2 million deaths occurred in 2012 worldwide [1]. A prime reason for the dismal clinical outcome of cancer is lack of effective management strategies for tackling tumor invasion and metastasis. Cancer metastasis is considered to be responsible for 90% of deaths associated with solid tumors [2]. However, till date, success in cancer treatment has been largely facilitated by early stage diagnosis and controlling localized cancer or primary tumor growth. This limited clinical success is further attenuated by the broad spectrum side effects of all the major anticancer drugs. Despite the extreme clinical significance of metastasis, therapeutic approaches to prevent or control advanced metastatic disease are presently in a very rudimentary stage [3, 4]. There have been numerous attempts by researchers to design drug that would interfere with various events of metastasis. For instance, bisphosphonates have proven efficacious in suppressing bone metastasis but they have not substantially improved overall survival [5]. Similarly, anti-VEGF monoclonal antibody Bevacizumab (Avastin) is a FDA approved therapy used to interfere with tumor angiogenesis. Despite its initially promising performance, anti-angiogenesis therapy is facing major challenges including inherent or acquired resistance, enhanced invasiveness during treatment [6, 7]. Integrin inhibitors (Abegrin, Vitaxin), Matrix Metallo Protease (MMP) inhibitors, Transforming Growth Factor β (TGFβ) inhibitors etc are being developed as a promising attempt to block invasion. However, unfortunately, either these inhibitors have not been efficacious in clinical trials [5] or have been found to cause an increase of tumor progression [8] or are too ubiquitous and hence non specific targets, which may cause intolerable side effects particularly in combination with standard therapies [9]. One of the main reasons for the poor therapeutic benefits of anti-metastatic treatments is probably the lack of therapeutic targets specifically aimed at controlling the pathologic course of metastasis [10]. This illustrates an urgent need to identify specific molecular ‘kingpins’ during the course of metastasis and also to develop agents which would target those molecules to control cancer spread.

In the past, research to identify molecular markers of tumorigenesis has largely focused on proteins. Although numerous protein biomarkers of cancer have been discovered and validated over the last few decades, a very few of these are being routinely used in the clinic [11]. Recently, increasing evidences point to the involvement of Long noncoding RNAs (lncRNAs) in a wide range of physiological processes [12]. Consequently, dysregulation of lncRNAs have been found to be involved in diverse human diseases including cancer [13]. Aberrant lncRNA expressions have been reported to be associated with tumor initiation, progression and metastasis of breast cancer, prostate cancer, colorectal cancer, lung cancer and so on [14]. For e.g., HOTAIR was found to be highly expressed in breast cancer samples [15] and MALAT1 was found to predict metastasis and survival in early stage non small cell lung carcimoma (NSCLC) [16]. Moreover, lncRNAs are very stable in body fluids and tissues increasing their prospects to be better biomarkers than proteins [17]. Additionally, given the pivotal role played by lncRNAs in cancer, targeting these molecules in cancer treatment is also considered to be a novel therapeutic approach [18]. Thus, identification of new lncRNAs involved in metastasis will not only serve as novel druggable targets but also provide more useful prognostic and diagnostic markers facilitating novel cancer treatment.

Here we report the discovery of a salt molecule of a bis Schiff base, M2 (Supplementary Figure 1A) as an anti-metastatic agent and also identify it's novel target, a long intergenic non-coding (LINC) RNA, LINC00273, for mediating its inhibitory effect on cancer cell migration and invasion *in vitro* and *in vivo*. For the first time we report a (4n-1) G quadruplex structure existing in the promoter of LINC00273 which is specifically targeted by M2 in the cancer cell to down regulate its expression. Furthermore, the real time expression of LINC00273 in thirty three human clinical samples is found to positively correlate with the histopathological staging of metastasis.

## RESULTS

### The molecule M2 and its cytotoxicity profile

M2 solubilizes in water and is doubly protonated at physiological pH [Supplementary Figure 1 (I) A-C]. The cytotoxicity of M2 was tested in normal human cells and also in an array of human and murine cancer cell lines at various concentration for 12hrs. The IC_50_ values revealed that M2 showed comparatively lesser cytotoxicity [Supplementary Figure 1 (I) D] unlike conventional cytotoxic anticancer drugs such as doxorubicin exhibiting narrow therapeutic index.

The antineoplastic effect of various doses of M2 was then investigated *in vivo* in three transplantable tumor models viz., Sarcoma-180 (S-180), Ehrlich ascites carcinoma (EAC) and B-16 melanoma [Supplementary Figure 1 (II) – (V)] & [Supplementary Figure 2 (I)]. The dose of M2 selected for further studies is 12.76 mg/kg b.w. per day (*i.e.,* 35μM) based on its higher therapeutic index. Detailed analysis of oxidative stress markers, liver function test, renal function test, haematogram [Supplementary Figure 2 (II)-(IV)] & [Supplementary Figure 3 (I)] and histopathological analysis of vital organs such as lung, liver and kidney signified the non-toxicity of the selected M2 dose towards the host. Repeated morphological and histopathological examination of the vital organs revealed a considerable reduction in the number of metastatic colonies in the organs of M2 treated tumor bearers than that of the untreated controls. In addition to these observations, the lower host toxicity of M2 prompted us to explore the effect of M2 on cancer cell migration and invasion.

### The antimetastatic potential of M2

**Figure 1:**
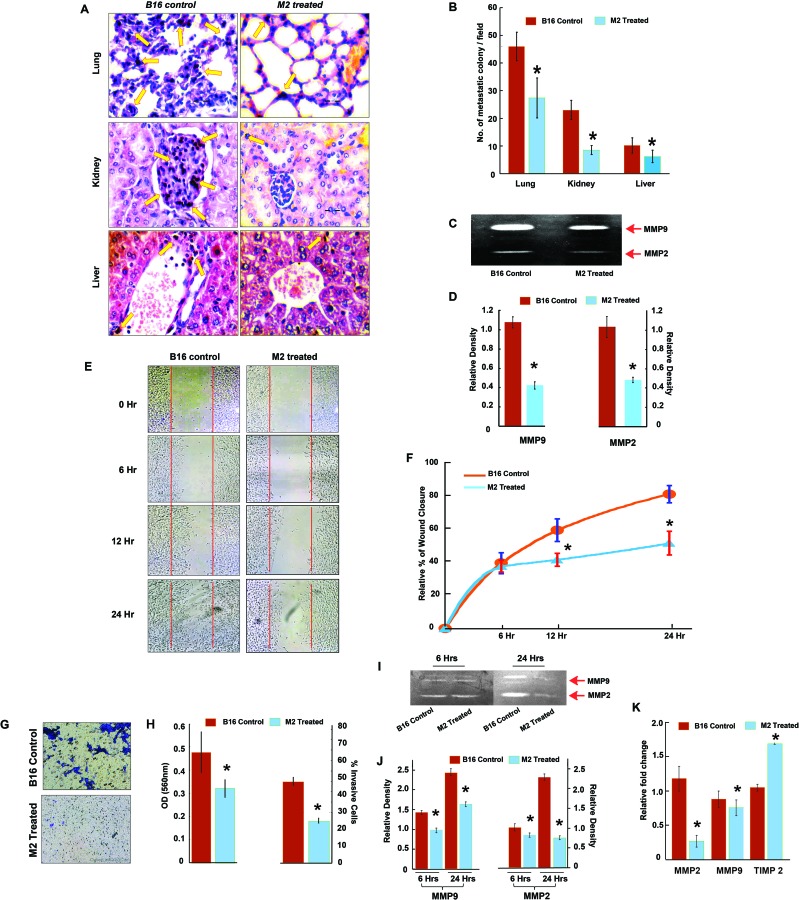
Anti-metastatic potential of M2 in murine melanoma, B-16F10 cells *in vitro* and *in vivo* **(A-B)** Histopathological analysis of lung, liver and kidney in M2 treated and untreated B-16F_10_ melanoma bearing C57BL/J mice. Representative H-E stained sections with arrows indicating metastatic colonies. **(C)** Effect of M2 on MMP activity analyzed by gelatin zymography. MMP-2 and MMP-9 activities were evidenced at the corresponding molecular weight. **(D)** densitometric analysis of MMP-2 and MMP-9 activities. **(E-F)** Wound-healing assay to assess the effect of M2 on cell B-16F_10_ melanoma cell migration at three different time points (6, 12 and 24 hours) after M2 treatment. **(G)** Effect of M2 on invasion ability was measured using transwell assays in B-16F_10_ melanoma cells. Microscopic observation (×10) of B-16 cells on the bottom of the Boyden's chamber at the end of the 24 h. **(H)** Optical Density (OD) and percentage of cells which have invaded to the lower chamber. **(I)** Effect of M2 on MMP activity analyzed by gelatin zymography. MMP-2 and MMP-9 activities were evidenced at the corresponding molecular weight. **(J)** Densitometric analysis of MMP-2 and MMP-9 activities. **(K)** qPCR data showing relative fold changes in gene expressions in M2 treated B-16 cells in comparison to untreated B-16 controls. (n=3 biological replicates, error bars denote s.e.m.). ^*^p value<0.005, relative to Control.

Anti-metastatic potential of M2 was initially explored in murine B-16F_10_ melanoma cell line for its added advantage of rapid growth and metastasis *in vivo* in C57BL/6 mice [19, 20]. Spontaneous metastasis of the B-16F_10_ melanoma cells to lung, liver and kidney of M2 treated and untreated tumor bearers was analyzed on day 21. Histopathological assessment of H-E stained sections of the organs suggested that M2 significantly decreased the number of metastatic colonies in these organs (Figure 1A & 1B). We further determined the activity of MMP-9 and MMP-2 which are considered to be most crucial markers in tumor invasion due to their ability to degrade the extracellular matrix (ECM) [21]. Activities of both the enzymes were found to be significantly decreased by M2 (Figure 1C & 1D).

In order to evaluate the effect of M2 on B16 cell migration an *in vitro* scratch wound assay model was used. A scratch was created on the 100% confluent monolayer of B16 and the effect of M2 on migration of B16 into the scratch was observed over a period 0, 6, 12 and 24 hrs. As shown in Figure 1E, at 0 hr, the scratch area created was devoid of any cells in both M2 treated and untreated wells. At 6 and 12 hrs, wells containing M2 showed 20±1.25% and 35±1.44% B16 cell migration in the scratch area, whereas in the untreated wells it was 38±3.25% and 57±4.14% respectively. Wound closure, at 24hrs, in M2 treated wells was 42±3.15% as compared to 75±5.2% in the untreated wells (Figure 1E & 1F). To further examine the invasive potential of B16 cells, a transwell system consisting of a Boyden's chamber coated with Matrigel was used. B16 cells were plated in the upper chamber and the percentage of cells moving to the underside of the coated membrane after 24 hrs was quantified using a microplate reader (Figure 1G & 1H). Results showed that there was almost 50% decrease in the number of cells that invaded the lower chamber after 24hrs of M2 treatment as compared to untreated cells. Inhibition of cell migration and invasion by M2 was accompanied by an increase in Tissue Inhibitor of Metalloprotease (TIMP) and decrease in mRNA levels and activities of MMP-2 and MMP-9 in B-16 cells (Figure 1I-1K).

**Figure 2:**
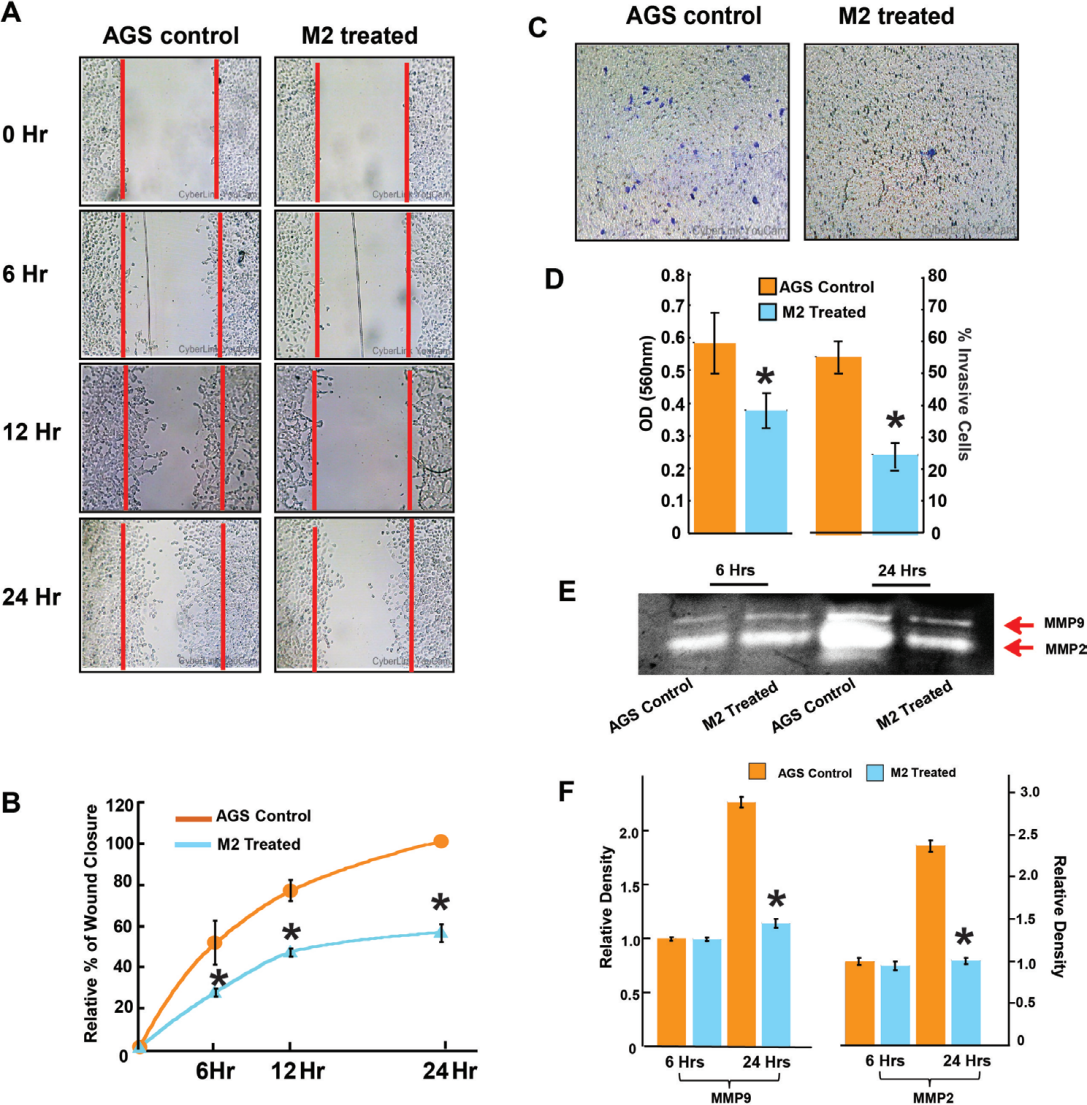
M2 inhibits human gastric cancer cell line AGS migration and invasion **(A-B)** Wound-healing assay to study AGS cell migration at three different time (6, 12 and 24 hours) after treatment with 35μM M2. **(C)** Effect of M2 on invasion ability was measured using transwell assays in AGS cells. Microscopic observation (×10) of AGS cells on the bottom of the Boyden's chamber at the end of the 24 h. **(D)** Optical Density (OD) and percentage of cells which have invaded to the lower chamber. **(E)** effect of M2 on MMP activity analyzed by gelatin zymography. MMP-2 and MMP-9 activities were evidenced at the corresponding molecular weight **(F)** densitometric analysis of MMP-2 and MMP-9 activities. (n=3 biological replicates, error bars denote s.e.m.). ^*^p value<0.005, relative to Control.

**Figure 3:**
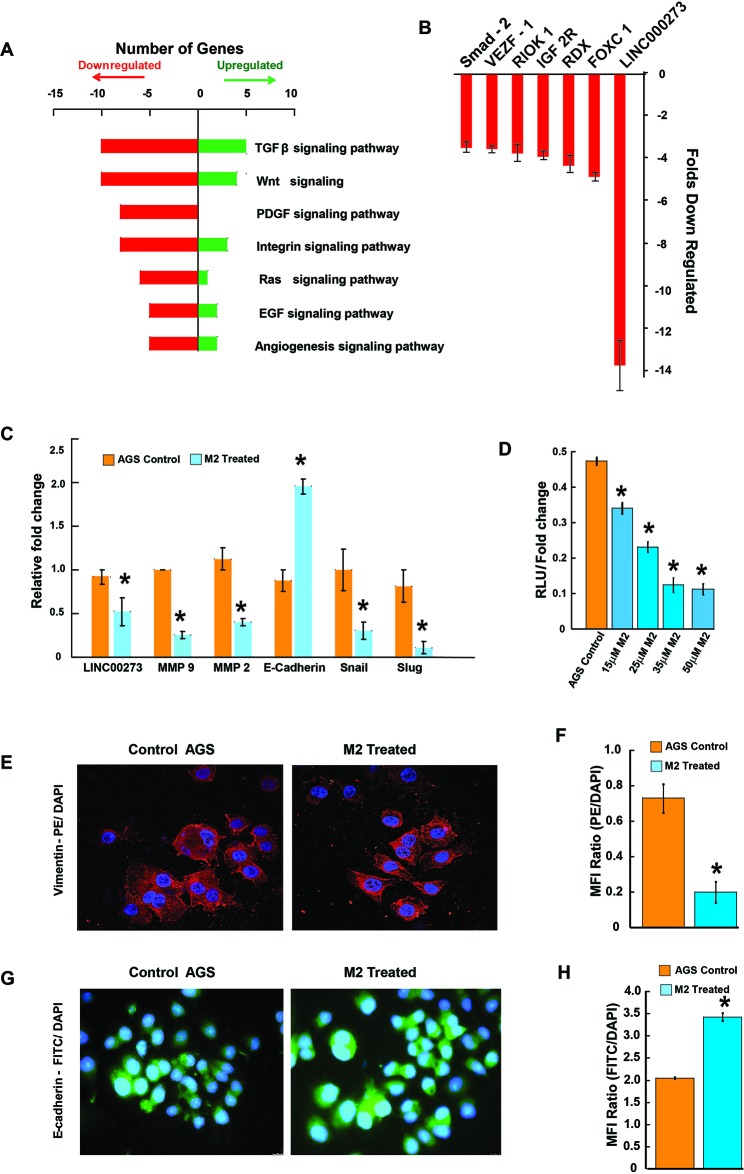
Tracing the molecules and pathways inhibited by M2 in preventing cancer cell migration and invasion **(A-B)** Graphical representation of microarray (Affymetrix) analysis showing some significantly upregulated or downregulated genes of important signaling pathways. **(C)** qPCR data showing relative fold changes in gene expressions related to cell migration and invasion in M2 treated AGS cells in comparison to untreated AGS controls. **(D)** Dual luciferase assay showing a dose dependent decrease in relative luciferase units indicating inhibition of LINC00273 transcription in presence of various concentrations of M2. **(E)** Representative fluorescent microscopic images of PE-tagged vimentin expression. **(F)** Bar diagram representation of the ratio of PE: DAPI Mean Fluorescence Intensity. **(G)** Representative fluorescent microscopic images of FITC-tagged E-cadherin expression. **(H)** Bar diagram representation of the ratio of FITC: DAPI Mean Fluorescence Intensity. (n=3 biological replicates, error bars denote s.e.m.). ^*^p value<0.005, relative to Control.

The migration-inhibitory role of M2 was next confirmed in a human gastric cancer cell line, AGS endowed with high metastatic potential. Results from scratch wound assay, conducted over a period of 0, 6, 12 and 24 hrs showed approximately 50% lesser closure of the scratch wound in M2 treated wells as compared to untreated wells (Figure 2A & 2B). M2 was also able to reduce the invasion of AGS cells by more than 50% as revealed by the transwell Boyden's chamber assay after 24 hrs of M2 treatment (Figure 2C & 2D). MMP activities and mRNA expressions were similarly downregulated in AGS (Figures 2E, & 3C). Having confirmed the anti-metastatic potential of M2, we next performed microarray (Affymetrix) to identify its probable molecular targets/pathways. Results revealed that M2 was able to downregulate many important signaling events in cancer progression such as EGF, PDGF, RAS, TGF-β, Wnt and Angiogenic pathways (Figure 3A), as well as some genes [22–26] such as Smad-2, VEGF, IGF, RIOK, FOXC1 etc [27–29] (Figure 3B) which are strongly connected to Epithelial to Mesenchymal transition (EMT), cell migration and metastasis. M2 downregulated several key mediators of EMT like snail, slug at their mRNA levels with concomitant elevation in E-cadherin transcription (Figure 3C) suggesting its potential to inhibit EMT, a prerequisite for cancer progression [30]. Expressions of mesenchymal marker, vimentin [Figure 3E-3F and Supplementary Figure 3 (III)] and epithelial marker, E-cadherin [Figure 3G-3H, Supplementary Figure 3 (II)], and was also found to be reduced at the protein level as measured by fluorescent microscopy.

According to the microarray analysis, amongst the various genes downregulated by M2, one particular LncRNA, LINC00273, drew our attention because it was downregulated ~14 folds following 12 hours of M2 treatment. This prompted us to investigate the involvement of LINC00273 in cancer metastasis and also to elucidate the interaction of M2 with this LINCRNA.

### A novel molecular target of M2: LINC00273

**Figure 4:**
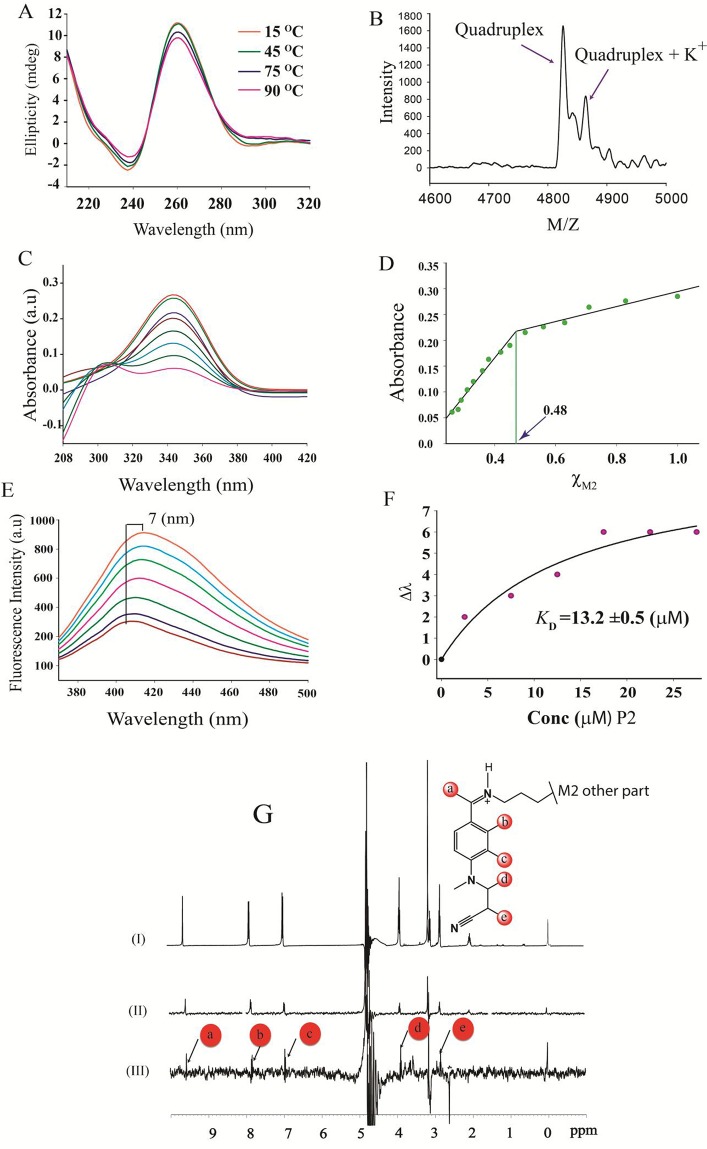
Spectroscopic Investigations **(A)** CD melting profile of P2-M2 complex. **(B)** MALDI-TOF spectra of P2 revealed intramolecular quadruplex formation. **(C)** Absorption spectra of M2 with increasing concentration of P2. **(D)** Job's plot analysis showed 1:1 complex formation for P2-M2 complex. **(E)** Fluorescence spectra of M2 with increasing concentration of P2. **(F)** Equilibrium dissociation constant (K_D_) calculated from the plot of Δλ (change in wavelength) vs. concentration of P2. **(G)** (I) One dimensional NMR spectrum of M2. (II) One dimensional reference Water-LOGSY spectrum of M2 and (III) Water-LOGSY spectrum of M2-P2 complex.

M2 has been described as a G-quadruplex targeting agent [31], therefore the significant decrease in the expression of LINC00273 by M2 treatment, prompted us to explore the presence of such a structure in the promoter of the said lnc RNA. Interestingly, we found two Quadruplex forming G rich motifs (i) P1, 5’- GGGCGGTGGGAGGGGGGTGGTGGG-3’ (ii) P2, 5’- GGGCGGGGAGGGGGG-3’) at the promoter of LINC00273 [Supplementary Figure 4 (IV) C&D). CD melting temperature (T_m_) of P2 and P2-M2 complex were found to be very high (>90°C) (Figure 4A) whereas M2 has no effect on the melting temperature of P1 [Tm of P1 and P1-M2 complex are comparable, ~79°C, Supplementary Figure 4(IV) A & B]. The Maldi-analysis of P2 identified intramolecular non-canonical G-quadruplex formation (Figure 4B) [32].

A sharp decrease in absorbance of M2 on increasing concentration of P2 in UV spectroscopy indicates ligand to receptor electronic transition which reinforces well stacking of M2 on the G-face of P2 (Figure 4C) [33]. As the transition dipole is proportional to the square of the molar extinction coefficients [34] at the buried state the dipole of the ligand is observed to be short. The Job's plot analysis confirmed 1:1 binding of M2 to P2 (Figure 4D). Fluorometric concentration dependent titration by P2 on M2 has shown a significant red shift [35] of 7 nm (dissociation constant, K_D_ was found to be 13.2 μM for P2-M2 complex formation) (Figure 4E-4F) indicating association of M2 with P2 [36]. The association of M2 with P2 was further proven by WaterLogsy ^1^H-1D NMR spectroscopy (Figure 4G).

However, absorption spectroscopy, fluorimetric titration analysis and WaterLogsy NMR showed that M2 does not bind to P1 [Supplementary Figure 4(IV) E and F; Supplementary Figure 4(V)].

In order to validate the interaction of M2 with the promoter of LINC00273, we assessed the effect of M2 on the promoter activity of the mentioned linc, by performing Dual Luciferase assay. Results showed a significant decrease in relative luciferase units for the plasmid constructs in presence of various concentrations of M2 (Figure 3D) as compared to untreated controls.

### The guanine-rich domain in the P2 promoter folds into G-quadruplex structure under physiological condition

**Figure 5:**
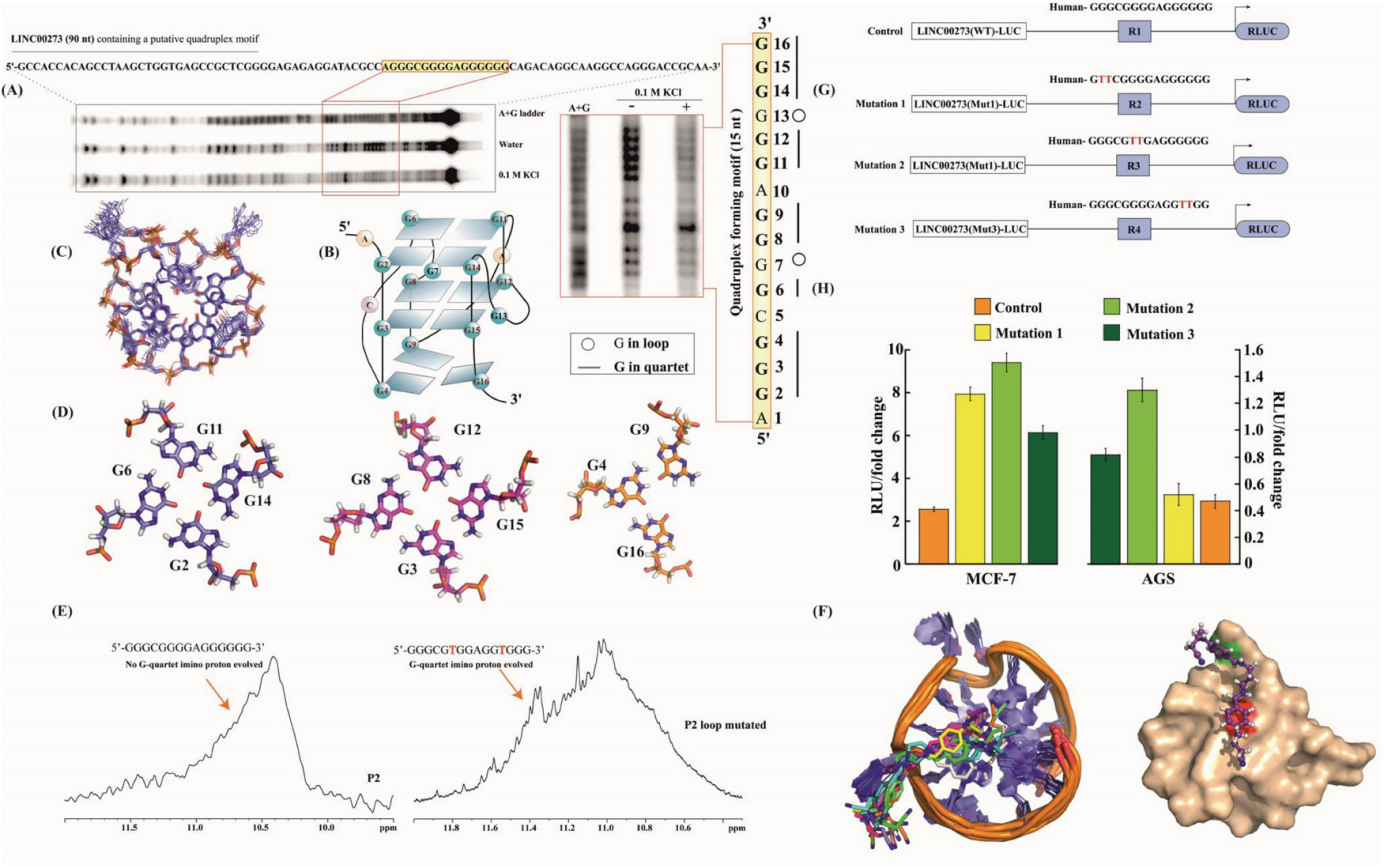
G-Quadruplex as a Target of M2 **(A)** DMS Footprint of 90 nt promoter region of LINC00273 DNA containing P2. **(B)** Schematic model of P2-G quadruplex structure obtained from DMS footprint. **(C)** Ensemble 10 structures of P2-G quadruplex from 100 ns MD simulation. **(D)** two G quartets and one triad forming P2 G quadruplex structure. **(E)** 1D-Imino proton spectrum of wild type P2-G quadruplex and 1D-Imino proton spectrum of mutated P2-G quadruplex. **(F)** Ensemble 10 structures of complex of M2-(P2-G quadruplex) from 100 ns MD simulationand surface structure of P2-G quadruplex bound to M2, red color denotes G11 and green denotes A10 in surface representation of P2. **(G)** Scheme and sequence of the putative M2 binding site (WT) and its mutations (Mut1 and Mut 2) within the LINC00273 promoter-reporter luciferase vectors [LINC00273(WT)-LUC and LINC00273(Mut1)-LUC & LINC00273(Mut2)-LUC]. **(H)** Dual Luciferase assay to show LINC00273 promoter activity in wild type (LINC00273(WT)-LUC) and mutated (LINC00273(Mut1)-LUC & LINC00273(Mut2)-LUC) vector constructs. There was a significant increase in luciferase activity of LINC00273(Mut)-LUC as compared to (LINC00273(WT)-LUC).

We performed dimethyl sulfate (DMS) protection assay to elucidate the core structure of G-quadruplex in the guanine-rich stretch of LINC00273 P2 under physiological conditions. The footprinting results show that the 15 nt motif in LINC00273 folds into a parallel quadruplex topology having two G-tetrads and a G-triad at the 3’ end constituting a 4n-1 core structure in contrast to other quadruplexes which conventionally harbours four guanines (4n) in the core. Appearance of a singular band in the electrophoretic mobility shift assay suggests intramolecular quadruplex in LINC00273 (Figures 5A and 4B). G6, G2, G3, G11, G13, G8, G12, G15, G14, and G9 are fully protected from DMS cleavage in 100 mM KCl suggesting them to be participating in tetrad formation. G7 and G13 are fully cleaved indicating their presence in the loop. Further analysis of the footprinting reveals that G6-G11-G14-G2 form the first quartet. G8-G12-G15-G3 form the second quartet and G16-G4-G9 form a triad in the third plane with a vacant G-site (Figure 5B-5D), same topological arrangement is maintained in the model structure. The vacant site may be occupied by a water molecule which coordinates with K^+^ ion and forms hydrogen bond with the guanines in the third plane to give structural stability. The flexible loop geometry and 4n-1 core structure provides a selective platform for interaction with M2. The in depth description of validation of structural integrity of the results of molecular dynamics simulation is included in the supplementary information. Molecular docking suggests that binding of M2 over 3′ end is more favorable than 5′ end binding of the (4n-1) G quadruplex structure [Supplementary Figure 5 (I)]. π-π stacking and CH_3_-π stacking interactions play dominant role in dictating the stabilization in binding of M2. RMSD analysis and B-Factor analysis of simulated trajectories [Supplementary Figure 5 (II)-(III)] suggests that presence of M2 at both the binding site has positive impact over the structural integrity of P2. Ensemble structures of last 1ns simulation steps of P2 in different states showed organized structural assembly of P2 with minimal deviations (Figure 5C). Water density map analysis [Supplementary Figure 5 (IV)] indicates that overall structure of P2 is rigid and binding of M2 is causing desolvation of respective binding site. Also, the stabilization of triad through structured water molecules is observed [Supplementary Figure 5(V)]. The energy parameters contributing to binding free energy of M2 bound at 5′ and 3′ end are enlisted in Supplementary Table 1. Interestingly, M2 binding at 5′ end is more stable than 3′ end. In the model structure (G2-G6-G11-G13) makes the first quartet at the 5’end, then (G3-G8-G12-G15) makes the second quartet and then G6-G4-G9 makes a stable triad at the 3’end (Figure 5D).^1^H NMR spectroscopy from the appearance of Hoogsteen hydrogen bonded ^1^H-imino signal of G quartets within the range of 10.2 ppm - 11.5 ppm [37]. The proton signal is broad because of fast conformational exchange of G-quadruplex scaffolds. This atomic resolution based evidence directly showed these two motifs are able to form G-quartet structures (data not shown).

As ^1^H spectrum P2 yields a broad imino signal we modified P2 (P2-loop mutated) with the sequence alternation 5'- GGGCG**T**GGAGG**T**GGG-3′ (according the analysis from DMS footprint) which yield sharp imino signals dictating slower conformational exchange in G quadruplex structure formation (Figure 5E). Ensemble structures of P2-M2 complex from the all atom molecular dynamics simulation strengthened the fact that structural integrity of M2-P2 complex is very high and A10 and G11 of P2 stack over the two benzene rings of M2 to stabilize the interaction (Figure 5F).

Mutations of conserved sites within LINC00273 promoter were used to further confirm the presence of G-quadruplex structure in the LINC00273 promoter (Figure 5G). Relative luciferase units were found to increase with respect to the control in AGS and MCF-7 cells transfected with mutant LINC00273 promoter construct (Mutation 1, 2 & 3 respectively) suggesting that disruption of the G-quadruplex structure increases the LINC00273 promoter activity (Figure 5G-5H). In addition, the M2-mediated inhibition in LINC00273 promoter activity (as shown in Figure 3D) was found to be compromised in AGS cells transfected with mutant LINC00273 promoter construct and exposed to M2 confirming that the binding of M2 to the G-quadruplex structure in the promoter of LINC00273 is essential for its inhibitory effect on LINC00273 (Supplementary Figure 6A).

### LINC00273 and its role in cancer metastasis

**Figure 6:**
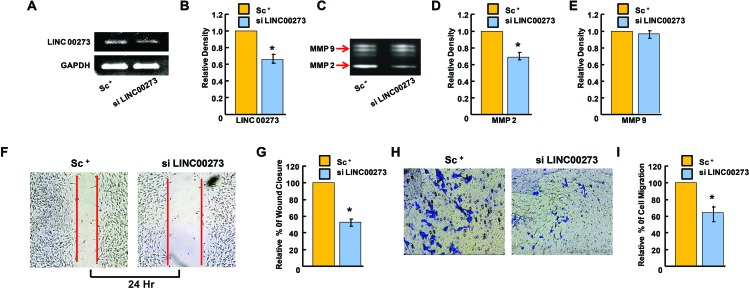
Effect of LINC00273 gene knockdown [using short interfering RNAs (siRNAs)] on B-16 melanoma cells **(A)** semiquantitative RT PCR expression of LINC00273 showing reduced expression in si LINC00273 treated B16 cells as compared to scramble control (sc+). **(B)** Densitometric analysis of LINC00273 expression measured by semiquantitative RT PCR showing efficiency of si RNA tranfection. **(C)** effect of siLINC00273 on MMP activity analyzed by gelatin zymography. MMP-2 and MMP-9 activities were evidenced at the corresponding molecular weight. **(D-E)** densitometric analysis of MMP-2 and MMP-9 activities. **(F-G)** Wound-healing assay to assess the effect of LINC00273 silencing on cell B-16 melanoma cell migration after 24hrs. **(H)** Effect of LINC00273 silencing on invasion ability was measured using transwell assays in B-16 melanoma cells. Microscopic observation (×10) of B-16 cells on the bottom of the Boyden's chamber at the end of the 24 h. **(I)** Optical Density (OD) and percentage of cells which have invaded to the lower chamber. (n=3 biological replicates, error bars denote s.e.m.). ^*^p value<0.005 relative to Scramble Control.

**Figure 7:**
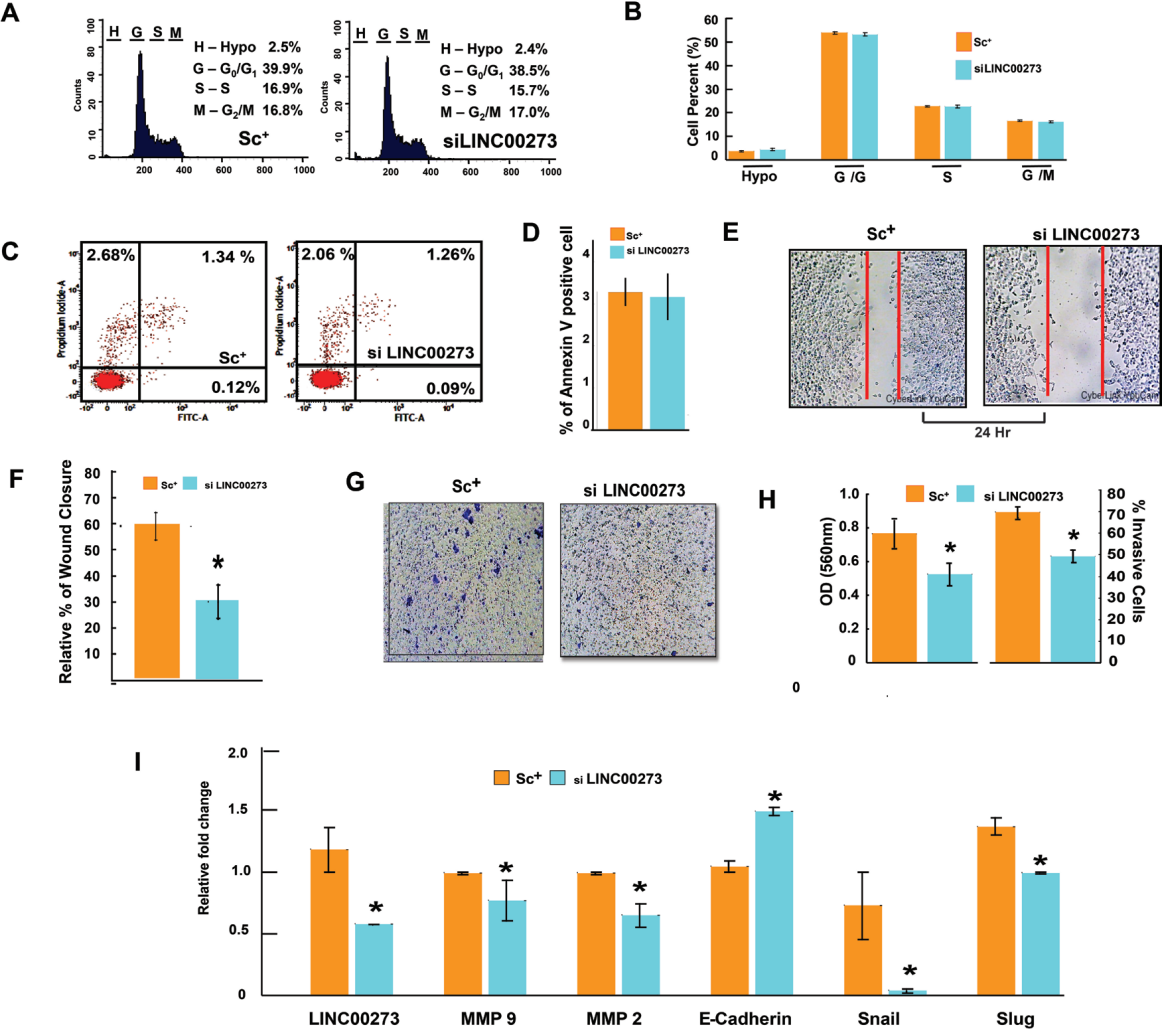
Effect of LINC00273 gene knockdown [using short interfering RNAs (siRNAs)] on AGS cells **(A)** Cell cycle phase distribution detected in a flowcytometer. Histogram display of DNA content (*x*-axis, PI-fluorescence) vs. counts (*y*-axis) has been shown. **(B)** Bar diagram representation of cell cycle phase distribution of scrambled siRNA control and si LINC00273 treated AGS. **(C)** FITC tagged Annexin V/PI Assay of AGS cells. Dual parameter dot plot of FITC-fluorescence (*x*-axis) vs. PI-fluorescence (*y*-axis) has been shown in logarithmic fluorescence intensity. In a double label system, scrambled siRNA control and si LINC00273 treated AGS cells were labeled with PI and Annexin V and analyzed on a Flowcytometer. **(D)** Bar diagram representation of percent Annexin V positive scrambled siRNA control AGS cells and si LINC00273 treated AGS cells. **(E-F)** Wound-healing assay to study AGS cell migration following si LINC00273 transfection. **(G-H)** Microscopic observation (×10) of AGS cells on the bottom of the Boyden's chamber at the end of the 24 h. Percentage of scrambled siRNA control and si LINC00273 treated AGS cells which have invaded to the lower chamber is shown graphically. **(I)** qPCR data showing relative fold changes in gene expressions related to cell migration and invasion in si LINC00273 treated AGS cells in comparison to scrambled siRNA control AGS. (n=3 biological replicates, error bars denote s.e.m.). ^*^p value<0.005 relative to Scramble Control.

**Figure 8:**
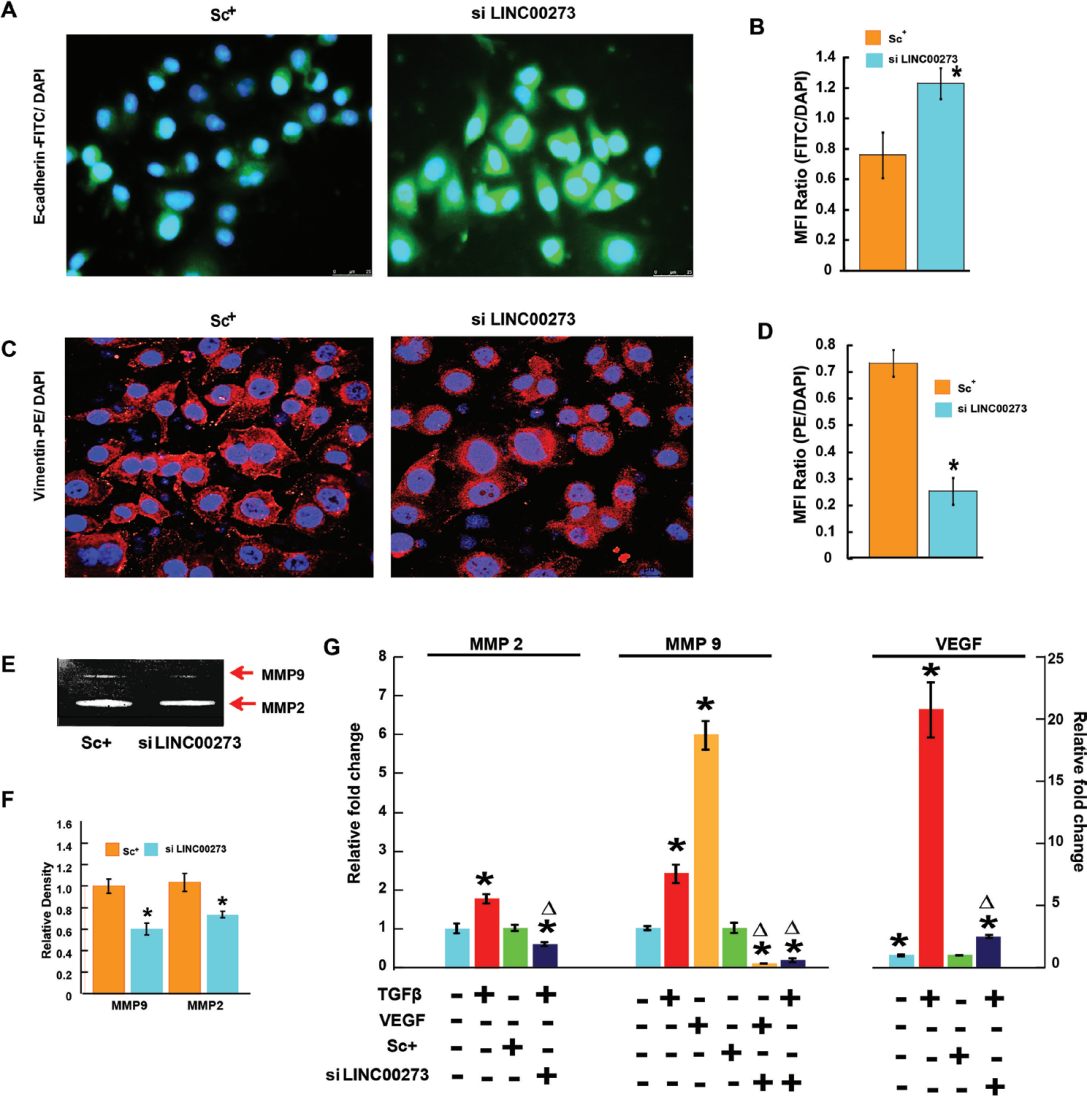
Tracing the changes of molecular expression related to angiogenesis and metastasis on silencing of LINCRNA000273 **(A)** Representative fluorescent microscopic images of FITC-tagged E-cadherin expression. **(B)** Bar diagram representation of the ratio of FITC: DAPI Mean Fluorescence Intensity. **(C)** Representative fluorescent microscopic images of PE-tagged vimentin expression. **(D)** Bar diagram representation of the ratio of PE: DAPI Mean Fluorescence Intensity. **(E)** effect of LINC00273 knockdown on MMP activity analyzed by gelatin zymography. MMP-2 and MMP-9 activities were evidenced at the corresponding molecular weight. **(F)** densitometric analysis of MMP-2 and MMP-9 activities in scrambled siRNA control and si LINC00273 treated AGS cells. **(G)** qPCR data showing relative fold changes in expression of MMP-2 and MMP-9 in the presence and absence of recombinant 10ng/mL TGF-β and VEGF in si LINC00273 treated AGS cells and scrambled siRNA control AGS. ^*^p value<0.005 relative Control. ^Δ^p value<0.005 relative to Scramble Control.

Having confirmed the binding of M2 with the LINC00273 promoter, we next investigated the role of this LINC-RNA in M2-mediated anti-metastatic activity. For this, we carried out transient knock down experiments in human and murine cancer cell lines. Small interfering RNA (siRNA) was used to knock down human LINC00273 in AGS cells and B16 melanoma cell lines. Non-targeting siRNA (known as scrambled siRNA) was used as control. Quantification analysis showed that LINC00273 expression levels were significantly knocked down by siRNA transfection. Cell migration was significantly attenuated in B16 melanoma cell line after siRNA mediated silencing of LINC00273 as evident from MMP activity, scratch wound assay and transwell cell migration in Boyden's chamber (Figure 6A-6I). Next, we carried out transient knock down experiment in AGS cell line and studied modulation, if any, of the cellular processes such as apoptosis, proliferation and metastasis. Cell cycle analysis performed after LINC00273 siRNA transfection in AGS cell line revealed no significant change as compared to scrambled siRNA transfected AGS cells (Figure 7A-7B). Similarly, Annexin V/PI assay in siRNA transfected AGS cell line also did not show any significant alterations. (Figure 7C-7D). However, the migration of AGS cells was significantly inhibited after transfection with LINC00273 siRNA as is evident from scratch wound and transwell migration assays (Figure 7E-7H). The significant downregulation in the real time mRNA expression of slug, snail with a concurrent upregulated expression of E-cadherin in LINC00273 silenced AGS cells point towards the role of this LINC in EMT (Figure 7I). Moreover, to further fortify the positive role of LINC00273 in EMT, we have shown that the protein level expression of E-cadherin (Figure 8A-8B) was significantly increased and that of vimentin (Figure 8C-8D) decreased in the absence of LINC00273. As shown in Figure 8E-8F, LINC00273 knock down markedly reduced the gelatinolytic activity of MMP-2 and MMP-9 produced from AGS cells. According to the micro array analysis, M2 significantly downregulated the TGF- β and VEGF signaling pathways (Figure 3A). Over expression of TGF-β and VEGF is positively correlated with metastasis and tumor aggressiveness. Both these factors are known to modulate the expression and activity of MMPs. Therefore, we next investigated the effect of LINC00273 knock down on MMP-2 and MMP-9 expressions upon TGF- β and VEGF stimulation. In our study, we have found that administration of recombinant TGF-β in AGS cells caused significant upregulation of MMP-2 & -9. Administration of recombinant VEGF in AGS cell line upregulated only MMP-9. Knocking down LINC00273 almost completely abolishes the sensitivity of AGS cells towards TGF-β and VEGF stimulation causing no upregulation of MMP-2 or MMP-9 (Figure 8G).

### Knocking down LINC00273 decreases AGS migration in nude mice

**Figure 9:**
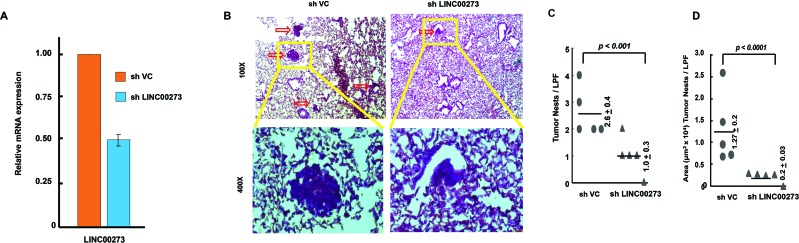
Xenograft model with stable knockdown of LINC00273 by shRNA in AGS cells **(A)** qPCR data showing expression of LINC00273 after shRNA mediated knockdown of LINC00273 (sh LINC00273) and comparing it with vector control shRNA (shVC). **(B)** The representative pictures of H&E stained lung sections from shVC and shLinc00273 AGS groups. **(C)** The number of lung tumour nests in each group was counted under a low power field (LPF). **(D)** Area occupied by the tumor nests per LPF is presented for sh VC and shLINC00273. Data represented as mean±s.d., ^*^P< 0.005 as compared to shVC.

Finally, to ascertain the role of LINC00273 in AGS cell migration *in vivo*, we performed stable knockdown of LINC00273 by using shRNA and injected into the tail vein of nude mice. As shown in Figure 9, knocking down LINC00273, decreased the extent of tumor cell migration to lung significantly. Histopathological analysis of H-E stained section of lung revealed the presence of metastatic cancer lesions in the lungs of the control group (shVC). Such metastatic lesions were found to be significantly lesser in the lungs of sh LINC00273 transfected mice (Figure 9B). These observations suggested that knockdown of LINC00273 could inhibit AGS cells metastasis *in vivo*.

### Expression of LINC00273 in clinical samples

**Figure 10:**
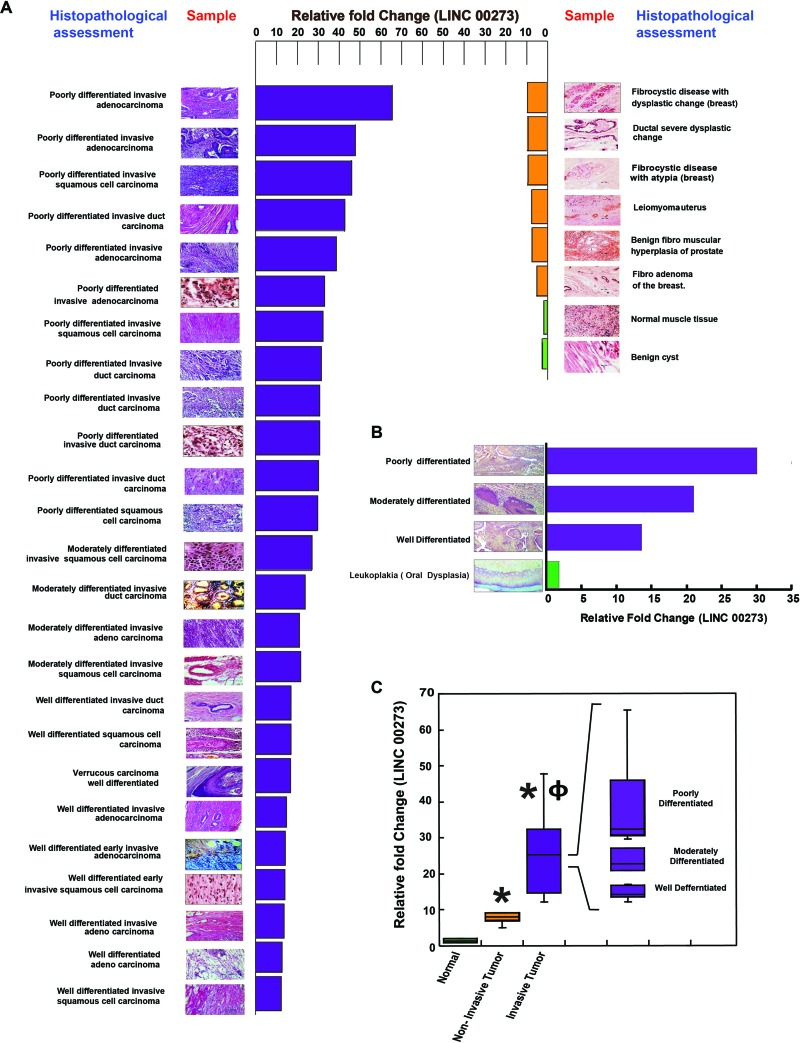
Correlation between histopathological assessment of clinical samples and their LINC000273 expressions **(A)** Data revealed distinct difference in expression of LINC000273 with varying stages/types of neoplasia. Normal scored minimum relative expression of the LINC00273 followed by benign tissue or carcinoma *in situ*. Invasive samples exhibited maximum LINC00273 with internal difference varying with well differentiated, moderately differentiated and poorly differentiated conditions. **(B)** Various stages of oral cancer, as identified histopathologically is correlated with the degree of LINC00273 expression. **(C)** Graphical expression of the difference of LNCRNA in accordance to samples being Normal, Benign and Metastatic. Data shows the expression of this LINCRNA can be used not only as a molecular target to restrict metastasis but also in the field of diagnosis / staging of the disease. ^*^p value<0.005 relative to Normal samples and ^ɸ^ p value<0.005 relative to Non-metastatic samples.

Ultimately, in order to confirm the positive role of LINC00273 in cancer metastasis, we studied its mRNA expression in a number of normal, benign and malignant clinical samples of various tissues such as breast, colon, prostate, intestine, uterus, oral etc. In order to corroborate our laboratory findings with clinical significance we randomly picked up thirty seven preprocessed clinical samples in the form of slides used for biopsy (Figure 10). Histological assessment done by a professional pathologist was correlated with Real time PCR expressions of LINC00273 (Figure 10A-10B) which suggested increased expression of this lncRNA with degree of metastasis. In terms of relative fold change, normal tissue (n = 2) scored least (< 2) followed by benign tissue or carcinoma *in situ* (n = 6) having features of hyperplasia or dysplastic changes without any signs of invasion, with scores lesser than 10. Clinical samples with histopathological interpretation relating to signs of invasion (n = 28) scored above 12. The relative scores in these malignant tissues could further be distinctly sub-grouped in accordance to varying degrees in terms of both invasiveness and differentiation. Well differentiated tumors with early signs of invasion exhibited LINC00273 fold changes that ranged between 12 to 17, while invasive tumors containing moderately differentiated cells ranged between 21 to 27 (Figure 10C). Poorly differentiated samples with invasiveness showed the highest relative fold changes in LINC00273 expression which is close to 30 and above. As an instance we have provided here various stages of oral cancer samples and have correlated LINC00273 expression level against traditional histological staging (Figure 10B).

This data proves the involvement of LINC00273 during cancer progression in various types of cancer. This study discovers LINC00273 as a novel inducer of metastasis whose knock down significantly inhibits EMT and metastasis.

## DISCUSSION

The failure of cancer therapies to treat malignant tumors has intensified the need for anti-metastatic drugs. In this study, we have shown that a salt molecule of a bis Schiff base, M2, despite being comparatively less cytotoxic to both cancer and normal cells as compared to conventional anticancer agents, was able to reduce tumor growth in three transplantable tumor models. This reduction in tumor growth was found to be mediated through inhibition of cancer cell migration and invasion by M2. The novel molecule has been shown to target several important signaling pathways including the TGF-β pathway which has profound pro-metastatic effects in advanced cancers such as EMT induction, angiogenesis, altered extracellular matrix deposition, immune suppression, and increased metastatic colonization (52). A number of downstream effector molecules of TGF-β like transcription factors (SNAIL and SLUG), growth factors (VEGF), matrix proteins and proteases (e.g., TIMPS and MMPs) are also regulated by M2. In addition, a very interesting observation was a 14 fold downregulation in LINC00273 expression in M2 treated cells as revealed by microarray analysis. In a previous study, we had reported M2 to be a G-quadruplex stabilizing agent and interestingly here we have found the presence of two quadruplex forming G rich motifs at the downstream coding region of LINC00273. Thus, the reduction in the expression of this lncRNA by M2 is proposed to be mediated by stabilizing the G-quadruplex structure present in its promoter. Given the anti-metastatic potential of M2 and the discovery of LINC00273 as its prominent target, we next explored the hitherto unreported function of this lncRNA. A novel finding of the present study is the involvement of LINC00273 in cancer cell migration and invasion. In this study our data showed that LINC00273 is frequently overexpressed in metastatic clinical tumor samples of different organs. To better evaluate the potential role of LINC00273 in AGS cell migration and invasion *in vitro* and *in vivo* in nude mice we knocked down the gene using siRNA and shRNA respectively. Results revealed that knocking down LINC00273, significantly decreased the migratory and invasive potential of AGS *in vitro* and *in vivo*. We have also shown that LINC00273 knock down makes the AGS cells unresponsive to TGF-β's pro-metastatic effects suggesting an important interaction between this LINC RNA and TGF-β signaling. Interestingly, the observed elevated expression of LINC00273 in tumor samples of various organs is found to be in increasing order from normal to benign to malignant implying that high expression of this linc may be a potential biomarker to detect cancer metastasis. A closer inspection of the histopathological interpretation and corresponding LINC000273 expression suggests that the expression of this LncRNA is correlated with degree of cellular de-differentiation which is an important step in tumor invasion. Samples from tissues marked as carcinoma *in situ* expressed equivalent amounts of LINC000273 as benign tissues which rules out LINC00273 as a marker of cancer per se. However LINC000273 expression in clinical tissues could be more prominently correlated with tumor histological staging characterized by differentiated, moderately differentiated and poorly differentiated conditions. This is indicative of the fact that, in all probability, the LncRNA in question participates particularly in EMT which is directly linked to enhanced migratory capacity and invasiveness. Thus, the expression of this LINCRNA can be used not only as a molecular target to restrict metastasis but also in the field of diagnosis / staging of the disease. Thus, M2 arrests a G-quadruplex structure in the promoter region of LINC00273 inhibiting its transcription which, in turn, seems to contribute to the anti-metastatic effect of M2. Furthermore, we have also conducted the pharmacokinetic parameter prediction, for M2 using GastroPlus^TM^ V.9.0 software. (Simulations Plus Inc., Lancaster, USA, US-FDA approved) (Suppl Ref 3), according to which M2 shows dose dependent bioavailability which confirms that the molecule is not significantly metabolised in the intestine and the liver. Although, initially at low dose volume M2 shows metabolism by CYP3A4 but in dose escalation study metabolism do not limit the bioavailability of M2. M2 has shown good solubility and permeability across the intestinal cell wall and hence can be classified as BCS (Biopharmaceutical classification system) class I, which pharmaceutical scientist always prefer. Additionally, absorption of M2 along the intestinal wall nullify the role of efflux transporters present on the apical membrane (Pharmacokinetic Analysis of M2 Supplementary, Page 19-23, Table pS1 and Table pS2).

**Figure:**
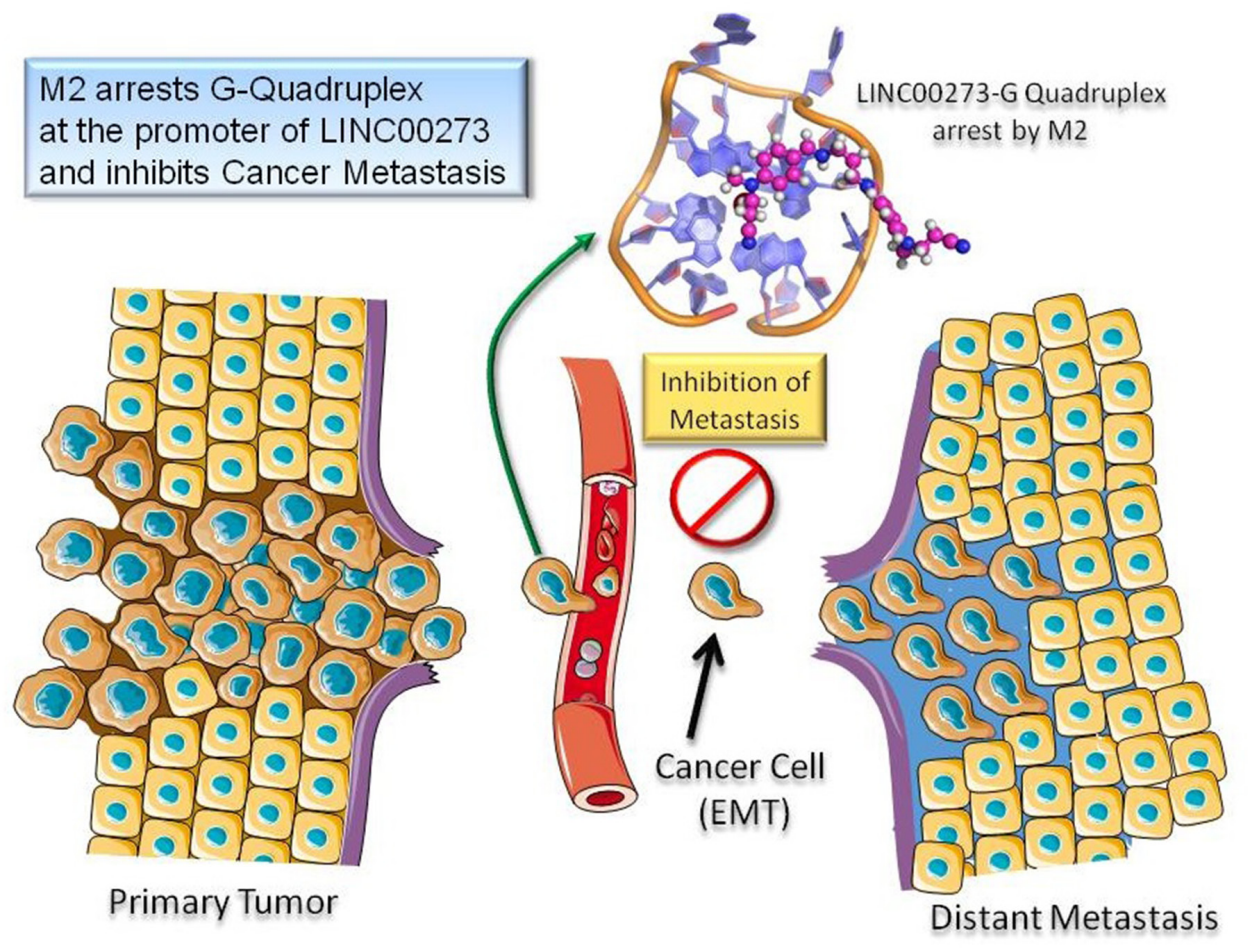
M2 inhibits cancer metastasis by binding to G-quadruplex structure at the promoter of LINC00273

These findings raise important questions that warrant future explorations. For example, it needs to be ascertained whether LINC00273 acts downstream or upstream of TGF-β signaling. The operation of TGF-β signaling in the absence of LINC00273 and *vice versa* also needs to explored in detail to elucidate their interdependence in mediating metastasis promoting effects. However, despite these and several other unexplored facets, the discovery of M2 as an anti-metastatic agent and the identification of LINC00273 as its target is a step forward towards formulating anti-metastatic treatment strategies in cancer.

## MATERIALS AND METHODS

### Chemicals and reagents

All reagents were purchased from Sigma (St. Louis, MO), unless otherwise indicated. Annexin V-FITC Kit was purchased from eBioscience (USA). siRNAs for LINCRNA00273 was procured from Dharmacon, USA. Power SYBR® Green Master Mix was obtained from Applied Biosystem, USA. Dual-Glo luciferase assay kit (Promega, USA). 24-well Chemicon® QCM™Invasion Assay kit was purchased from Millipore, Billerica, USA. 1,3-diaminopropane and 4-[2-Cyanoethyl)methylamino]benzaldehyde, tolune were purchased from Sigma Aldrich company for the synthesis of M2.

### Animal models for solid tumors

Male C57BL/6 and Swiss albino mice were maintained in plastic cages (~6 mice / cage) at an ambient temperature of 22-25°C on a 12 hour light / dark cycle with access to drinking water and pellet diet National Institute of Nutrition (NIN, Hyderabad, India) *ad libitum*. All animals used were procured as per authentication from NIN, Government of India, Hyderabad, Andhra Pradesh, India. During experiments the age of all animals were ~ 5 months bearing body weight ~ 27gms. Use of animals was under strict animal care ethics guidelines of the Institute. Animal experiments were repeated three times per set. All animal experiments were done following the institutional ethical guidelines approved by the committee.

### Solid tumor production

The murine B-16F10 melanoma cells were maintained *in vitro* and Sarcoma-180 cells were maintained in male Swiss albino mice. Solid tumors were produced by subcutaneous inoculation of 1×10^6^ B-16 F10 and S-180 cells on the dorsal surface of right hind leg of C57BL/6 and Swiss albino mice respectively. Viability was assessed by the Trypan blue dye exclusion method.

### Treatment

After seven days of tumor inoculation, different concentrations of M2 (15-40μM) at doses 5.47mg/kg, 9.119mg/kg, 10.943mg/kg, 12.76mg/kg, 14.5mg/kg body weight was administered intraperitoneally every alternate day for 21 days after tumor inoculation.

### Experimental groups

All the animals were randomly divided into following groups of 6 animals each: i) saline treated normal mice ii) M2-treated (at various doses) normal mice iii) Melanoma control iv) Sarcoma Control v) EAC control vi) M2-treated (at various doses) Melanoma bearing mice vii) M2-treated (at various doses) Sarcoma bearing mice viii) M2-treated (at various doses) EAC bearing mice. The weights of all the animals belonging to different groups were recorded weekly throughout the experimental period.

### Sera isolation

Blood was collected from the tail vein. Serum was separated by allowing blood to clot at a slanting position for 45mins then centrifugation at 1,500 × *g* for 30 min at 4°C. Finally, serum samples were stored in aliquots at −20°C for later use.

### Measurement of serum biochemical parameters

Alanine aminotransferase (ALT), aspartate aminotransferase (AST), alkaline phosphatase (AP), urea and creatinine levels were measured from collected sera using Autospan liver function test kit, Span Diagnostics Ltd., Surat, India.

### Total WBC count

Counting chambers that hold a specified volume of diluted blood (Haemocytometer) were used to calculate the number of red and white cells per litre of blood.

### Measurement of tumor volume and tumor weight

The antitumor activity was assessed by measuring tumor weight and the changes in tumor volume. Changes in tumor size over time after tumor transplantation was assessed in all the experimental groups. The length and width of the tumor were measured using calipers. Tumor volume was calculated by the following formula:

Tumor volume (mm^3^): 0.5 × a × b^2^ where a is the largest diameter and b its perpendicular.

### Dissection and tissue collection

All the mice were euthanized after the last dose of M2 treatment. Liver, lungs, kidneys and tumor tissues of the animals from all the experimental groups were collected, washed in 0.9% saline, soaked in filter paper and processed for cellular, biochemical and histological studies. Femurs were also aseptically removed; bone marrow was flushed with a 26-gauge needle and suspended in RPMI for cell count and cell cycle analysis.

### Histopathological assessment

Tumor, liver, lungs and kidney tissues were fixed overnight at 4°C in freshly prepared 4% paraformaldehyde and then dehydrated in graded alcohols and embedded in paraffin. Sections of 5μm thickness were cut from representative paraffin blocks. Tumor tissues were cut right through the middle of the tissues to obtain the central core region. The sections were rehydrated and stained with hematoxylin and eosin (H&E). Stained sections were observed under light microscope (Olympus CH20*i*).

### Assay of hepatic lipid peroxidation (LPO)

The extent of LPO in the bone marrow and liver homogenates was determined quantitatively by performing the method as described by Ohkawa et al., 1979 [38]. The amount of malondialdehyde (MDA) was measured by reaction with thiobarbituric acid at 532 nm using spectrophotometer (Eppendorf BioSpectrometer Kinetic). MDA levels were calculated using the standard curve of MDA and its level expressed in nM/mg of protein.

### Assay of anti-oxidant enzymes

#### Catalase (CAT) activity

CAT activity was assessed by the method of Luck [39], wherein the breakdown of H_2_O_2_ is measured. Briefly, assay mixture consists of 3 mL of H_2_O_2_ phosphate buffer and 0.05 mL of the supernatant of the tissue homogenate. The change in absorbance of the reaction mixture was recorded for 2 min at 30 s interval at 240 nm using a spectrophotometer.

### Superoxide dismutase (SOD) activity

SOD activity was assayed by the method of Marklund et al.[40] based on pyrogallol auto-oxidation inhibition and expressed as unit/mg of protein. One unit of enzyme activity is defined as the amount of enzyme necessary for inhibiting the reaction by 50%. Auto-oxidation of pyrogallol in Tris-HCL buffer (50 mM, pH 7.5) was measured by increase in absorbance at 420 nm using a spectrophotometer.

### Glutathione-S-transferase (GST) acitivity

GST activities were measured in tissue cytosol by determining the increase in absorbance at 340nm with 1-chloro-2,4-dinitrobenzene (CDNB) as the substrate and the specific activity of the enzyme was expressed as formation of CDNB-GSH conjugate per minute per milligram protein [41].

### *In vivo* nude mouse metastasis model

This study was performed at Vivo Bio Non Clinical Research Services (VBNCRS), C/o. Vivo Bio Tech Ltd, Sy. No: 349/A, Pregnapur Village, Gajwel Mandal, Siddipet District, Telangana, India - 502311. The study has been approved by the IAEC of the test facility which is approved by CPCSEA with registration number 1117/PO/RcBiBt/S/07/CPCSEA. Female NCr Nude mice (4-6 weeks) were maintained under specific pathogen-free conditions at VBNCRS. All experimental procedures involving animals were undertaken in accordance with the test facility's guidelines. For the tumor metastasis model, 1×10^6^ Lenti-shVC and Lenti-shLinc00273 stably infected AGS cells were injected into tail veins of mice (n=5 per group) and then were kept under observation for 21 days. All the mice were anaesthetized and sacrificed. Livers were excised and fixed followed by H&E sections.

### Cell lines and culture

Passage 11 of Human gastric cancer cell line (AGS), Passage 15 of Human breast cancer cell line (MCF-7) and Passage 9 of murine B-16 melanoma cell line were obtained in 2014 as authenticated from national cell repository situated at National Centre for Cell Sciences (NCCS), Department of Biotechnology, Government of India, Pune, Maharashtra, India. The cells, initially seeded at the concentration of 10^4^cells/mm^2^ were cultured at 37°C in humidified atmosphere of 5% CO_2_–95% air. AGS cells were cultured in DMEM and F12K (Gibco, invitrogen, USA) whereas MCF-7 and B-16 melanoma were cultured in RPMI 1640 supplemented with 10% heat inactivated fetal bovine serum (FBS; Invitrogen.) and 1% penicillin G and streptomycin (Life Technologies, Rockville, MD, USA). *Mycoplasma* contamination detection was done using PlasmoTest (Invivogen. USA), all experiments were performed in *Mycoplasma* free cell lines.

### Cell count

Cells from tumor tissue were harvested and a single cell suspension was prepared in RPMI supplemented with 1nM EDTA and collagenase (200*μ*g/mL). As the core regions of solid tumors contain necrotic zones, the peripheral regions were chosen for making single cell suspension. The viable tumor cells were counted in a haemocytometer by the Trypan Blue exclusion method.

### Cell cycle distribution analysis from *in vitro* and *in vivo* samples

After making a single cell suspension, cells were fixed with 3% p-formaldehyde, permeabilized with 0.5% Triton X-100, and nuclear DNA was labeled with propidium iodide (PI, 125*μ*g/mL) after RNase treatment using. Cell cycle phase distribution was determined on FACS Calibur using Cell Quest Software (Becton-Dickinson Histogram display of DNA content (x-axis, PI fluorescence) vs. counts (y-axis) has been displayed. Cell Quest statistics was used to quantitate the data at different phases of the cell cycle.

### Detection of apoptosis from *in vitro* and *in vivo* samples

Apoptosis assays were carried out based on the instruction from the Annexin V Apoptosis Kit (Biovision, Milpitas, CA). Briefly, PI and Annexin V Fluos were added directly to the single cell suspension of the tumor tissue. The mixture was incubated for 15min at 37°C. Cells were fixed and then analyzed on FACS Calibur (equipped with 488 nm Argon laser light source; 515 nm band pass filter, FL1-H, and 623 nm band pass filter, FL2-H) (Becton Dickinson, San Jose, CA).

### Monolayer wound healing assay

Cells were grown in 35 mm plates. After the cells reached sub-confluence, the monolayer cells were wounded by scraping off the cells with sterile 200μL pipette tip across the center of the well and then grown in medium for 6h, 12 and 24hrs. Digital images of the cells were captured at 0, 6, 12 and 24 hrs after wounding. Wound area was measured by the program Image J (http://rsb.info.nih.gov/ij/). The percentage of wound closure was estimated by the following equation: Wound closure % = [1-(wound area at Tt/wound area at T_0_) x100%, where Tt is the time after wounding and T_0_ is the time immediately after wounding.

### Invasion assays

Invasion of M2 treated and untreated cells was analyzed using the Chemicon® QCM™ 24-well Invasion Assay kit (Millipore, Billerica, USA) according to manufacturer's instruction. Invaded cells on the bottom of the insert membrane were stained with 400μL of cell stain after 48 hours. The stain insert was transferred to a clean well containing 200μL of extraction buffer. After 15minutes 100uL of dye mixture was transferred to a 96-well microtiter plate for colorimetric measurement at 560nm.

### Detection of gene expression by RT-PCR

Total RNA from AGS and B-16 cells was isolated using TRIzol (Applied Biosystems/Ambion, Austin, TX, USA). RNA from clinical samples (obtained upon requisite approval from the Human Ethical Committee of Barasat Cancer Hospital, Kolkata, India) was isolated using PureLink™ FFPE RNA Isolation Kit Invitrogen, USA) according to the manufacturer's instruction. Concentration and purity of the isolated RNA was measured spectrophotometrically at 260 nm and 280 nm.

Reverse transcription was performed using MMLV Reverse Transcriptase (NEB, UK). A mix of 2μg total RNA and 2μL of random primers (NEB, UK) was incubated in a total volume of 15μl for 5 min at 70°C and cooled on ice. After adding 5 μL of MMLV 5× Reaction Buffer (NEB, UK), 10mM dNTPs, 1μL ribonuclease inhibitor (NEB, UK) and 2μL of MMLV Reverse Transcriptase to reach a total volume of 25μL, the reaction mix was incubated again for 1h at 45°C.

PCR reactions were performed using Hot Start Taq 2X Master Mix (NEB, UK) in reaction mixes containing 10nM of each primer, 1–4μL cDNA, and PCR buffers as supplied by the manufacturer, in a total volume of 25μL.

PCR primers used for the analysis (shown in Table 1) were designed based on sequences deposited in the Primer 3 database: Products were run on 1% agarose gel and visualized by ethidium bromide staining.

**Table 1:** Primer sequences of the genes investigated in RT-PCR analysis

	No.	Primer	Sequence
Human	1	LINC00273(L)	GCCACACAGTAGGTGACGAG
		LINC00273(R)	ACTGCTTTCGGGAGAGAATG
	2	MMP2(L)	AAGGGCATTCAGGAGCTCTA
		MMP2(r)	TCCTGTTTGCAGATCTCAGG
	3	MMP9(L)	CCGGACCAAGGATACAGTTT
		MMP9(R)	CGGCACTGAGGAATGATCTA
	4	E-cadherin(L)	TTCCAGGAACCTCTGTGATG
		E-cadherin(R)	TCTTGGCTGAGGATGGTGTA
	5	Snail(L)	CTAGGCCCTGGCTGCTAC
		Snail(R)	GACATCTGAGTGGGTCTGGA
	6	Slug(L)	CTGGCCAAACACAAGCAG
		Slug(R)	ACCCAGGCTCACATATTCCT
	7	GAPDH(L)	ATCATCCCTGCCTCTACTGG
		GAPDH(R)	GTCAGGTCCACCACTGACAC
	8	Foxc1(L)	GGACAAGGAGGAGAAGGACA
		Foxc1(R)	AAGCCGATCCTCTGTGACTC
	9	VEZF1(L)	ACTTGCAGTGTTTGTGGGAA
		VEZF1®	GTTTGGCATTTGAAGGGTCT
	1	MMP2(L)	GGAGATCTGCAAACAGGACA
		MMP2(r)	AACCGGTCCTTGAAGAAGAA
Mouse	2	MMP9(L)	TCCTTGCAATGTGGATGTTT
		MMP9(R)	CGTCCTTGAAGAAATGCAGA
	3	GAPDH(L)	ACAACTTTGGCATTGTGGAA
		GAPDH(R)	GATGCAGGGATGATGTTCTG
	4	TIMP2(L)	CCCAGAAGAAGAGCCTGAAC
		TIMP2(R)	GGAGGAGATGTAGCAAGGGA

Real time PCR was performed using Power SYBR® Green Master Mix. Quantitative RT-PCR was run using an sds7500fast Sequence Detection System. Data were analyzed with Singleplex (Applied Biosystems, CA). Each PCR was performed in triplicate at least 3 times independently.

### Microarray analysis

The M2 treated and untreated AGS cells were lysed with TRIzol ^®^ reagent (Life Technologies, US) and total RNA was extracted according to the manufacturer's protocol. The RNA samples were then sent to iLife Discoveries (Gurgaon, India) to perform microarray analysis (Affymetrix GeneChip ^®^ Human Primeview Genome Array). Each group sample set was comprised of three replicates. The Affymetrix GeneChip ^®^ system was used for hybridization, staining, scanning, and imaging of the arrays. Raw data were separately analyzed using Expression console, R-Bioconductor (Limma) & Affymetrix TAC software. One-way analysis of variance (ANOVA) was performed with GeneSpring 7.31 software (Agilent, Santa Clara, CA, USA) to identify genes whose expression changed significantly between the M2 treated and control AGS cells.

#### Clustering for differentially expressed genes

The unsupervised hierarchical clustering of 170 genes regulated in 6 hr vs. C, 90 genes in 12 vs. C & 370 genes in 12hr vs. 6hr were then analyzed in Genesis tool followed by hierarchical clustering algorithm. The gene symbols and normalized data (log2 ratio) was uploaded in Genesis software for all three compared condition. The clustering heat map image signifies that treated sample 6 & 12 hrs have very different type of expression pattern and genes comes under this group have very different expression pattern.

### Small interfering RNA and transfection

Small interfering RNAs (siRNAs) targeting LINC000273 were designed and synthesized by Dharmacon (Thermo Fisher Scientific Biosciences, Colorado, USA). We have selecting the pool of four siRNA for the knockdown of LINC00273. The siRNA sequences were as follows: 1: Sense: 5'-G.A.G.A.G.A.U.U.U.C.C.A.A.U.G.U.U.U.C.U.U-3'

Antisense: 5′ 5′-P.G.A.A.A.C.A.U.U.G.G.A.A.A.U.C.U.C.U.C.U.U-3′

2: Sense: 5′-C.C.G.A.G.C.U.C.C.U.G.U.G.G.U.U.U.C.A.U.U-3′

Antisense: 5′-U.G.A.A.A.C.C.A.C.A.G.G.A.G.C.U.C.G.G.U.U-3′

3: Sense: 5′-A.A.A.G.C.A.G.U.G.A.G.G.U.G.G.A.U.G.G.U.U-3′

Antisense:5′-P.C.C.A.U.C.C.A.C.C.U.C.A.C.U.G.C.U.U.U.U.U-3′

4: Sense: 5′-A.G.A.G.A.U.U.U.C.C.A.A.U.G.U.U.U.C.C.U.U-3′

Antisense: 5′-5′-P.G.G.A.A.A.C.A.U.U.G.G.A.A.A.U.C.U.C.U.U.U-3′.

For transfection, B-16 and AGS cells were seeded in 24-well plates and transfected with 5μL (50nM) siRNA using a Lipofectamine™ 2000 reagent (Invitrogen). Each experiment was done in triplicate and at least three times independently.

### Establishment of shRNA mediated stable knockdown of AGS cells

Three interfering shRNAs were selected to target linc00273 and expressed using the pLKO.1-puro (promega, USA) vector. The shRNA sequences were as follows: 1: 5′ -CCGAGCTCCTGTGGTTTCATT-3′ 2: 5′-AAAGCAGTGAGGTGGATGGTT-3′ 3: 5′-AGAGA TTTCCAATGTTTCCTT-3′. The Lentivirus Packaging of shRNA cloned plasmid were done in the packaging cell line 293T HEK (5 × 10^6^). 1 × 10^5^ AGS cells were seeded in each well of a 12-well plate in 500 μL of complete media and transduced by lentiviral vectors at a multiplicity of infection of 10:1. Stable clones were selected and grown for one week in complete media containing 8 μg/mL puromycin (Sigma Chemicals, St Louis, MO, USA) [42].

### Site-directed mutagenesis

Site-directed mutagenesis was used to mutate the putative M2 binding sites in the LINC00273 promoter of the construct LINC00273(WT)-LUC. It was performed by overlapping PCR using a commercial QuickChange II Site-Directed Mutagenesis kit (Agilent Technologies, USA).

The PCR primers used for mutagenesis was Forward:and the resulting construct was denoted LINC00273(mut)-luc. The sequence accuracy of all constructs was confirmed by DNA sequencing and the construct was used in luciferase reporter assays.

### Reporter assay

For the reporter gene assay, The LINC00273 promoter region (CCAGTGGCCCGTGGCAGCGCCACACAAGGCGGAGCCGGGTTTGGTCCCAGAGGGGGCCACCACAGCCTAAGCTGGTGAGCCGCTCGGGGAGAGAGGATACGCCAGGGCGGGGAGGGGGGCAGACAGGCAAGGCCAGGGACCGCAAGGGCAAGGGCACCCGGGAGCCCGCAGAGGGGCGGCTCGGGAAGAAACCTCAGGCAAAGCCGGGCCACCAGGAAAACACGGCCACGGGATCCCACCGCCACAGATACGAGGGAGGTCCCGCGGCGCCCCGCCTAGGAAGCCGGACGGCCCCTGGCACCCACCGAGACCCGCCTCACGAGCCTGGGTCCCGCCATCGGGACCCCGAAGCGACCTCAGCCACAAACCCAACGCCAGGGCCACGTTGCTGGTTTCTTGTCCATCCTCCGACACTGTCAAGCTCCGGGAGACCGGCGCGCCCCCCACTTGG) was cloned into the pGL4.72 vector containing the Renilla luciferase reporter gene. AGS cells were cultured in 24-well plates for 24hrs and transfected with 200 ng of the construct, along with 20 ng of the TK promoter cloned vector containing the firefly luciferase gene (BioBharati LifeScience Pvt.Ltd, India). The transfection was carried out using 2μL of lipofectamine 2000 according to the manufacturers protocol (Gibco Life Technologies, Gaithersburg, MD, USA). Cells were harvested 48hrs after transfection and lysed using 1 × passive lysis buffer (Promega, Madison, WI, USA). Luciferase assays were performed as described by us previously [43] and using the Dual-Glo luciferase assay kit (Promega, USA). The ratio of *Renilla* over firefly luciferase activities was calculated and considered as the final Relative luciferase activity value. Each assay was performed in triplicate and repeated at least three times.

### Gelatin zymography

Metalloproteinase activity was analyzed by gelatin zymography. B-16 and AGS cells were cultured and treated for the last 24 hr of culture with M2 (35 μM) in serum free medium. This treatment period did not alter final cell number as determined by trypan blue exclusion (not shown). The supernatant volume was electrophoresed in 10% SDS polyacrylamide gels containing 1 mg/mL gelatin. Gels were then incubated in 2.5% Triton X-100 to remove SDS, washed briefly in distilled water and then incubated in 50 mM Tris–HCl, 10 mM CaCl_2_ (pH 7.5) overnight at 37°C. The gels were then stained with 0.25% (w/v) Coomassie brilliant blue and destained with 10% isopropanol in 10% acetic acid. The gelatinolytic activity was identified as transparent bands in the blue background.

#### DMS protection assay

The 90 nt long LINC oligonucleotides are purchased from Xcelris Genomics Pvt. Ltd which encompasses the 15 nt long guanine rich stretch flanked by the sequences of Chromosome 16 (NCBI ref. seq NC_000016.10). The 5′ ends of the oligonucleotides are radiolabeled by [γ -32P] ATP using T4 polynucleotide kinase (Fermentas) in ultrapure DNase/RNase free distilled water (Invitrogen) and are passed through Microspin G-25 columns (GE-Healthcare) to remove the unincorporated radioactive γ-32P as per manufacturer's instructions and further purified in 16% native PAGE. The radiolabelled DNA is recovered from the gel by overnight incubation in PAGE elution buffer and ethanol-precipitated. The annealing is followed by heating the samples at 90°C for 5 min and then cooling slowly to room temperature in 20 μL of 10 mM Potassium-phosphate (pH 7.0) buffer (10 mM K_2_HPO4 + 10 mM KH_2_PO4) with or without 100 mM KCl. The samples are then methylated by treatment with 0.1% (final concentration) DMS for 2 min at room temperature. The reaction was stopped by stop solution (0.3 M sodium acetate (pH 7.0) and 1M Beta-mercaptoethanol), and subsequently ethanol-precipitated. Methylated DNA samples are subjected to cleavage with 1 M piperidine for 20 min at 95°C. The samples are lyophilized to remove the piperidine and three successive water washes. The cleaved products (2500 cpm/5 μL) are dissolved in formamide dye and analysed on a 10% sequencing (denaturing) urea-PAGE gel containing along with A+G sequencing ladder of the same 90 nt long DNA fragment. The gel is dried and autoradiographed using Typhoon Trio+ phosphorimager (GE Healthcare) to visualize the resolved DNA fragments.

### Synthesis

1,3-diaminopropane (1equivalent) was added drop wise to the solution of compound 1 (2 equivalent) in tolune at room temperature in presence of catalytic amount of con.HCl. The reaction mixture was allowed to stir at room temperature for 12 hour. The product was extracted in dichloromethane. Dichloromethane was removes under reduced pressure. The structure was confirmed NMR spectroscopy. Melting point of M2 was 96±2°C.

### NMR spectroscopy

All NMR spectra were recorded using Bruker AVANCE III 500 MHz NMR spectrometer equipped with a 5 mm SMART probe at 298K. NMR samples were prepared in water containing 10% D_2_O. DSS was used as an internal standard (0.0 ppm). One dimensional proton spectra of M2 were recorded using “zgesgp” with a spectral width of 15 ppm and number of scan 128. One dimensional ^13^C spectra of M2 were recorded using “zgdc” pulse programme with a spectral width of 237 ppm. Two dimensional HSQC and HMBC spectra of M2 were recorded using “hsqcetgpsi2” and “hmbcgplpndqf” respectively. Water LOGSY NMR experiment was carried out as per our previous paper [31]. ^1^H NMR(500 MHz): δ 7.63(1H, d, J=8.77 Hz), 6.71(1H, d, J=7.63Hz), 2.6(2H, t, J=13.69 Hz), 3.76(2H, t, J=13.69Hz), 3.64(2H, t, J=13.08 Hz), 2.073(2H, m, J=13.08 Hz), 3.09(3H, s), 8.17(1H, s).

### HPLC

All the compounds were purified using a reverse phase HPLC system (Shimadzu, Japan) using the phenomenex C18 column (dimension 250×10mm) by linear gradient elution technique using a dual solvent system (water and methanol, 0-80% CH_3_OH in 40 min).

### Circular dichroism spectroscopy

The secondary conformation adopted by P1 and P2 were determined using Jasco 815 spectrometer. The spectra was scanned over a range of 220 -310 nm using 0.1 cm path length cuvette and data interval with an average of three scans was 1 nm at room temperature. CD melting studies of P1/P2 and P1-M2/P2-M2 complexes (1:3 ratio) were recorded from 15°C to 90°C with Ramp rate of 2.5°C/min and temperature interval of 5°C. All spectra were base line corrected.

### Fluorescence spectroscopy

Hitachi spectrophotometer (F-700 FL spectrophotometer) were used for Fluorescence experiments. 0.1 cm path length cuvette was used. The spectra was scanned over a range of 370-500 nm with excitation wavelength at 345 nm fixing the excitation and emission slit at 5 nm. The scanning speed was 240 nm/sec with a response of 2.0 s. K_d_ (dissociation constant) was calculated from binding curve obtained from the plot of Δλ versus concentration of P1/P2 added using the equation.

$$f=B_{\max}*\text{abs}(x)/K_{d}+\text{abs}(x))$$ (1)

where Kd is the dissociation constant, x is the concentration of P1/P2, f is the change in wavelength and B_max_ is the maximum specific binding in the same units as f.

### UV spectroscopy

UVstudies were carried out using Hitachi U-2910 spectrophotometer. The spectra was scanned over a range of 250-420 nm keeping a data interval of 0.5 nm with a scanning speed of 200 nm/min. JOB plot was used to determine the binding stoichiometry between M2 and P1/P2. The absorbance at 345 was plotted against mole fraction of M2 in M2-(P1/P2) complex. The binding ratio is obtained using the following equation:

$$M2:P1/P2=\chi_{M2}:(1-\chi_{M2})$$ (2)

where χ_M2_ the mole fraction of M2 in M2-(P1/P2) complex.

### Structure building, molecular docking and molecular dynamics simulation in AMBER

The biophysical analysis suggests that promoter region ‘P2’ of LINC gene forms a stable secondary structure of parallel stranded G-quadruplex. Structural details of quadruplex formed in P2 at the atomic level are difficult to obtain due to its dynamic behavior. DMS foot printing data and mutational analysis using NMR spectroscopy offered the topological indexing of bases which are present in the loop regions and bases involved in the guanine stacking. Based on these observations we built the model structure of quadruplex for P2.

Model structure was built over the template structure (2M27) [31]. The stacking arrangement of guanines was retained from the template structure whereas loop regions were built using maestro [44]. Primary minimization of model structure was performed in macromodel [45] module of maestro with 2500 minimization cycles. AMBER^*^ forcefield was applied with water as a solvent system. Force constrained was applied over the stacked guanines while loop bases were minimized with PRCG method. Minimized structure was further employed for simulation run. Energy stabilized model after 100 ns of simulation run was further used for molecular docking of M2. Feasible conformations of M2 were generated with LigPrep [46]. P2 structure was refined with protein preparation wizard [47]. GLIDE [48] module and the extra standard precision mode (XP) of docking was used with 10 Å inner grid and 30 Å outer grid. As the binding site for M2 was not known, initially we performed the blind docking i.e. entire structure of quadruplex was taken for generating the grid. All the docked conformations were found to be localized at the 3'end, thus we further built a grid precisely over 3′ region. M2 was docked again with XP mode. To avoid the structural limitations of docking due to rigid nature of receptor and ligand and to investigate the possible binding of M2 at 5′end we imposed constrained docking after generating grid at 5′ site. Thus the simulation analysis was performed for M2-P2 complexes one at 3′ and one 5′ bound state.

Standard simulation protocol was followed as per our previous studies [49]. Quadruplex system was parameterized with FF14SB [50–53], other fragments were parameterized with GAFF [52]. M2 was parameterized using Antechamber module of AMBER14 [51, 52]. Whole systems were neutralized with Na^+^ ions and further solvated with 8 Å TIP3PBOX water model. Systems were minimized with two step minimization, further heated to 300K for 50 ps, and equilibrated for 1 ns. Prepared systems were further employed for 10 ns production run (NPT ensemble) at 300 K temperature and 1 atm pressure, with a step size of 2 fs. Temperature and pressure was maintained with Langevin thermostat, barostat and vibration of bonded hydrogen atoms was treated with SHAKE algorithm [54, 55]. Long range electrostatic interactions were calculated with Particle Mesh Ewald method [56] with 0.1 nm grid space of fast Fourier transform grid and the non-bonded cutoff was kept at 8 Å. Trajectories were analyzed with Ptraj module of AMBER, and visualized using PyMol and VMD [57–59].

### Statistical analysis

The experiments were repeated three times and the data were analyzed statistically. Values have been shown as standard error of mean. Data were analyzed and one-way ANOVA was used to evaluate the statistical differences. Statistical significance was considered when P< 0.05.

## SUPPLEMENTARY MATERIALS FIGURES, TABLES AND REFERENCES



## Abbreviations

LINC RNA: Long Intergenic Non Coding RNA
EMT: Epithelial to Mesenchymal Transition
BCS: Biopharmaceutical classification system
DMS: Dimethyl Sulphate
TGF-β: Transforming Growth Factor-beta
IARC: International Agency for Research on Cancer
FDA: Food and Drug Administration
MMP: Matrix Metallo Proteases
VEGF: Vascular Endothelial Growth Factor
LNC RNA: Long Non Coding RNA
EAC: Ehrlich ascites carcinoma
S-180: Sarcoma-180
ECM: Extracellular Matrix
TIMP: Tissue Inhibitor of Metalloprotease
EGF: Epidermal Growth Factor
PDGF: Platelet Derived Growth Factor
RMSD: Root Mean Square Deviation
NMR: Nuclear Magnetic Resonance
siRNA: Small Interfering Ribonucleic Acid
shRNA: Short Hairpin Ribonucleic Acid
PI: Propidium Iodide
H-E: Haematoxylin Eosin
CYP 3A4: Cytochrome P450 3A4
ALT: Alanine aminotransferase
AST: Aspartate aminotransferase
AP: Alkaline phosphatase
LPO: Hepatic Lipid peroxidation
MDA: Malondialdehyde
CAT: Catalase
SOD: Superoxide Dismutase
GST: Glutathione-S-Transferase.
