# Supplementary material for: LINCRNA00273 promotes cancer metastasis and its G-Quadruplex promoter can serve as a novel target to inhibit cancer invasiveness
Source: Oncotarget. 2017 Nov 17;8(66):110234–56. doi: 10.18632/oncotarget.22622 (PMC5746379; doi:10.18632/oncotarget.22622)
Supplement: Supplementary file 1 [file oncotarget-08-110234-s001.pdf]

LINCRNA00273 promotes cancer metastasis and its G-Quadruplex promoter can serve as a novel target to inhibit cancer invasiveness

SUPPLEMENTARY MATERIALS

The detailed analysis of molecular modeling study and MD simulation

Molecular docking analysis

Molecular docking suggests that binding of M2 over 3' end is energetically more favorable as blind docking resulted into localization of all the docked poses over the said area (Supplementary Figure 5(I)A). Thus constrained docking was performed over 3' end (Supplementary Figure 5(I)C) to get more precise bound conformation. In the docked conformation at 3'end, benzene ring of M2 is stacking over G16 (3'end), G10 is forming hydrogen-π interactions with benzene ring of M2, at the same time M2 is forming CH_3-π interactions with A1 (5' end). Further, constrained docking over 5'ends was performed to explore it as another feasible binding site (Supplementary Figure 5(I)B). In the docked conformation at 5'end benzene ring of M2 is stacked over G11 further; M2 is forming hydrogen-π interaction with G6. Docking scores (Supplementary Figure 5(I), Table 1) of constrained docking suggest that 3' site is favored by M2.

Analysis of simulated trajectory

Correlation of properties analyzed below with the structural features of respective subject is described in detail in our previous studies [1, 2].

Also, the mathematical details behind each calculation are same as mentioned in our previous studies [1, 2].

RMSD analysis

Backbone RMSD (Supplementary Figure 5(III) A) suggests that P2-LINC is stable in unbound as well as M2 bound state. Binding of M2 at 3' end imparts no significant variation in the overall RMSD however binding of M2 at 5' end results into lowering of backbone RMSD. Guanines involved in first G-stack formation (G2, G6, G11, and G14) posses lower RMSD upon M2 binding, P2-LINC in complex with M2 at 5' end has stable most first G-stack (Supplementary Figure 5(III)B). Second G-stack of P2-LINC is equally stable in all the systems, RMSD of respective bases (G3, G8, G12, G15) is unaltered in bound and unbound state. It indicates that ligand binding at 3' or 5' is not affecting the conformation of guanines present in second G-stack (Supplementary Figure 5(III)C). RMSD of guanines present in third G-stack (G4, G9, G16) is slightly increased by presence of M2 (Supplementary Figure 5(III D). RMSD of M2 (Supplementary Figure 5(III) E) is low for entire simulation run except for short simulation span where M2 is showing increased flexibility. Thus, RMSD analysis suggests that presence of ligand rigidifies the stacked guanines and overall backbone structure is strengthened further. Though presence of M2 at both the binding site has positive impact over structural integrity of P2-LINC, M2 bound to 5' end is found to be more effective.

The *B-factor analysis*

Loop regions and bases present at end terminals are showing flexible nature and guanines involved in stacking arrangement are forming rigid core (Supplementary Figure 5(II)). Further description is in comparison to the flexibility of unbound state. Presence of M2 is increasing the flexibility of A1 (5'); the effect is evident when M2 is bound at 3' end. Presence of M2 is increasing the flexibility of G16 (3') to small extent when M2 is bound at 3' end. Flexibility of Loop-1 and Loop-2 is decreasing when M2 is binding at 3' end however it is increasing when M2 is binding at 5' end. Loop-3 is least flexible and binding of M2 is rigidifying the region. Binding of M2 at 5' end is stabilizing the Loop-4.

Water density map analysis

Water density map analysis (Supplementary Figure 5(IV)) indicates that overall structure of P2-LINC is compact and rigid as the surrounding water structure is dense in all the three systems however loop regions are desolvated due to their flexible property. Binding of M2 is causing desolvation of respective binding site; M2 binding at 5' site is more solvated than 3'. Thus we can state that secondary structure of P2-LINC itself is highly rigid and binding of M2 maintains the structural integrity.

At 3' end of P2-LINC quadruplex, instead of tetrad there is a formation of guanine triad with one vacant position. Water density map analysis suggests the stabilization of triad through structured water molecules (Supplementary Figure 5(IV)). When M2 is bound at 3' end, water molecules are concentrated in the central region within the close proximity of G16 (H1 atom), Also H22 of G16 is solvated, water is bridging between the Watson-crick face of G4 and Hoogsteen face of G9, Watson-crick face of G9 is also solvated. In unbound state similar water structure is observed however solvation around H22 of G16 is lacking. When M2 is bound at 5' end water molecules are localized around H1 atom of G16

and Watson-crick face of G9 is also solvated however it is deficient in rest of the solvation pattern.

Conformational analysis

M2 when bound at 5' end, forms strong π-π stacking interactions with G11 (first G-stack) and G13 (Loop-4). At 3' end, M2 is highly mobile as at a time it is forming π-π stacking interactions with any of the two guanines of third G-stack thus roaming over 3' face (4, 9, and 16) during the simulation run. M2 forms H-π interactions with A1 while stacking over with G16 and G9 thus locking A1 near 3' end. The positioning of M2 over G16 and G9 facilitates H-π interactions of central hydrogen atoms of M2 with G4. Stacking of M2 over G4 and G9 retains the H-π interactions with A1.

In unbound state, A1 (5' end base) is folding inward towards 3' end, binding of M2 perturbs the stacking of A1. In 5'-end bound M2 complex, A1 is maintaining the stacked conformation for significant span while for certain time period it adopts the extended form and again fold back over 3' end. As described earlier, in 3'-end bound M2 complex A1 is locked over 3' end due to ligand mediated interactions however as 5' end is unoccupied A1 first gets extended and further get rearranged over 5' end.

Loop-1 (C5) and Loop-2 (G7) are positioned outward and are stacking with each other, in unbound state of P2-LINC this stacking arrangement is fragile thus breaking in between, in 5'-end complex stacking arrangement is stronger than unbound state however it is broken towards the end of the simulation, in 3' end complex the stacking arrangement of Loop-1 and Loop-2 is retained throughout the simulation run. Loop-3 (A10) is facing out in all the three systems, in 3' end complex it is providing binding site for M2. Loop-4 (G13) folds back over 5' end in unbound state, in 5'-end complex it is providing binding site for M2 and in 3'-end complex G13 is interacting with A1 thus locking it over 5' end region.

Binding free energy estimation

The energy parameters contributing to binding free energy of M2 bound at 5' and 3' end are enlisted in Supplementary Figure 6B. Binding of M2 is energetically favorable at both the sites as ΔG_{MMGBSA} and ΔG_{MMPBSA} are in negative range. As MMGBSA energy estimation assist in ranking of binding phenomenon, here it is found that M2 binding at 5' end is more stable than 3' end. Also, energy contribution due to van der Waals interactions and electrostatic interactions is in support with 5' end binding of M2. Thus free energy estimation indicates that binding of M2 is energetically satisfactory and its binding is more inclined towards 5' end.

In summary, the theoretical results coordinate with the experimental findings; M2 is binding with P2-LINC majorly through aromatic interactions. Both 5' and 3' faces are found to be potential sites for M2 binding, loop bases are further assisting the complex formation.

REFERENCES

1. Kaulage M, Maji B, Bhat J, Iwasaki Y, Chatterjee S, Bhattacharya S, Muniyappa K. Discovery and structural characterization of G-quadruplex DNA in human acetyl-CoA carboxylase gene promoters: its role in transcriptional regulation and as a therapeutic target for human disease. J Med Chem. 2016; 59: 5035-5050.
2. Bhat J, Chatterjee S. Skeleton selectivity in complexation of chelerythrine and chelerythrine-like natural plant alkaloids with the G-quadruplex formed at the promoter of c-MYC oncogene: *in silico* exploration. RSC Adv. 2016; 6: 36667-36680.

PHARMACOKINETIC ANALYSIS OF M2

INTRODUCTION

Pharmacokinetics (PK) prediction is a crucial phase of drug discovery pipeline. A prior prediction of PK parameters increases the clinical prevalence of molecules [1]. The commonly considered PK parameters for the orally administered compounds include clearance (CL), volume of distribution at steady state (V_{ss}), the fraction absorbed (fa), the rate of absorption (ka) and bioavailability (F). The single point calculation of these parameters is feasible practically and can help to prioritise the candidates for future development, but they do not unveil significant information about the dynamic behaviour of the compound concentration. Therefore, the *in silico* techniques for the prediction of PK prediction is highly desired [2]. GastroPlus (Simulations Plus, Lancaster, CA) is a software package for PK simulation. The prediction of PK parameters is mechanistic and is based on the database of human and animal physiological parameters [3].

In this work, the *in silico* PK modeling studies were performed to predict the human PK parameters of M2, using its molecular properties. The results have demonstrated good PK profile for the molecule under study.

METHODS

PK modeling using gastroplus™

The PK parameter prediction was performed for M2 using GastroPlus™ V.9.0 software (Simulations Plus Inc., Lancaster, USA) [3]. It uses a physiologically-based nine compartment model, where each compartment corresponds

to the various segments of digestive tract. For the prediction of PK parameters, various molecular properties of M2 were used (Table pS1). Using the ADMET predictor module of GastroPlus^{TM}, various compound relevant PK parameters were predicted for M2. Simulation of drug absorption was performed in Single Simulation Mode of Human-Physiological-Fasted physiological model (with default Absorption Scale Factor (ASF) Opt logD SA/V 6.1 parameters), at a dose of 100 mg and 200 mg, followed by an immediate release of M2. Parameter Sensitivity Analysis (PSA) was performed using GastroPlus^{TM} built-in PSA simulation mode, to identify the parameters critical for oral absorption. The various parameters such as dose, particle size, reference drug solubility and effective human jejunal permeability, were varied in

a range of one tenth to ten-fold of the tested input values. The impact of parameter variability was ascertained on bioavailability.

RESULTS

Human PK modeling for M2

The PK prediction, in human for M2 was performed using *in silico* techniques. The predicted values of various PK parameters are shown in Table pS2 and Figure pS1. The predicted values for 100 mg oral IR tablet show absorption of 99.87% and bioavailability of 19.89%, indicating a rapid and complete absorption of M2 and a reduced drug bioavailability, possibly due to first pass metabolism as it is predicted to be a substrate of CYP3A4. To understand the regional distribution of absorption, advanced compartmental absorption and transit (ACAT) model was used. The results are depicted in Figure pS2, which shows that almost complete absorption will occur in the small intestine. The predicted results are in accordance with the known optimum absorption site *i.e.* small intestine. The highest percent absorption was predicted to be from jejunum1 (40.9%). Duodenum and jejunum2 were predicted to absorb 30.7% and 15.8% of M2. Further the same study is repeated by increasing the dose to 200 mg; we find out that as the dose of the drug is increased the Cmax and bioavailability of the drug also increased to 23.92% and 0.44 µg/mL respectively. The absorption and the regional absorption remains same as of 100 mg. The PSA was performed to evaluate the influences of change in various molecular parameters on fraction bioavailability, fraction absorbed and C_{max}. The results suggested that a change in dose have the maximum influence on the studied parameters (Figure pS3). Thus, it can be concluded that an increase in the administered dose will improve the C_{max} as well as the percentage absorption and bioavailability of the drug.

CONCLUSION

M2 has shown the dose dependent bioavailability which confirms that M2 is not significantly metabolised in the intestine and the liver. Although, initially at low dose volume M2 shows metabolism by CYP3A4 but in dose escalation study metabolism do not limit the bioavailability of M2. M2 has shown good solubility and permeability across the intestinal cell wall and hence can be classified as BCS (Biopharmaceutical classification system) class I, which pharmaceutical scientist always prefer. Additionally, absorption of M2 along the intestinal wall nullify the role of efflux transporters present on the apical membrane.

REFERENCES

1. Van De Waterbeemd H, Gifford E. ADMET in silico modelling: towards prediction paradise? Nat Rev Drug Discov. 2003; 2: 192-204.
2. Ekins S, Waller CL, Swaan PW, Cruciani G, Wrighton SA, Wikel JH. Progress in predicting human ADME parameters in silico. J Pharmacol Toxicol Methods. 2000; 44: 251-272.
3. GastroPlus TM, Lancaster CA. In Lancaster, Unites States of America. Available online: http://www. simulations-plus.

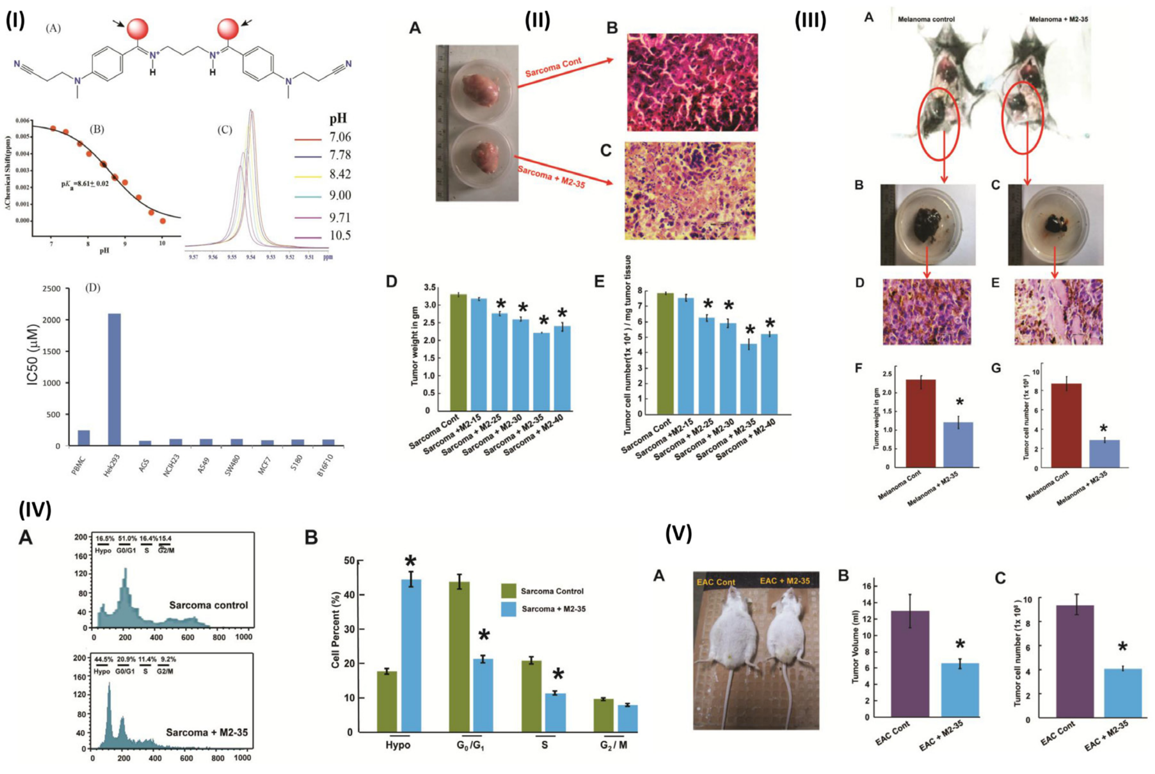

Supplementary Figure 1: (I) (A) Chemical structure of M2-protonated (functional form). (B) pK_aprofile of imine nitrogen. (C) One dimensional NMRspectra of marked hydrogen atom (red ball) at diffferent pH. (D) IC_{50} values of M2 at different Cancer cell lines.:Antitumor activity of M2 in S-180 tumor model. **(II)** (A) Representative photographs of tumors from treated and untreated mice to show change in tumorsize (B) & (C) Micrograph of representative H-E stained sections of tumortissue. (D) Change in tumor weight. (E) TumorcellCount. The data represented as mean+S.D. for the three different experiments performed in triplicate. Significant difference ($p<0.05$) is indicated with the following symbols“*”when compared with Sarcoma control.S-180 tumor cell cycle phase distribution detected in a flowcytometer. **(III)** (A) Histogram display of DNA content (x-axis, PI-fluorescence) vs. counts(y-axis) has been shown. (B) Bar diagram representation of cell cycle phase distribution of S-180 from different experimental groups. The data represented as mean+S.D. for the three differentexperiments performed in triplicate. Significant difference ($p<0.05$) is indicated with the following symbols“*”when compared with Sarcoma control. Antitumor effect ofM2 against EAC tumor model. **(IV)** (A) Representative picture of the untreated EAC bearing mice compared with treatment with M2 (B) Treatment with M2 was able to significantly reduce Tumor volume. Mice were sacrificed and peritoneal fluid was measured in measuring cylinder 4 weeks after intraperitoneal injection of EAC cells. (C) Tumor cell count. Significant difference ($p<0.05$) is indicated with the following symbols“*”when compared with EAC control. Antitumor effect ofM2 against B-16 melanomatumor model. **(V)** (A) Representative picture of the untreated melnoma bearing mice compared with treatment with M2 (B)& (C)Representative photographs of tumors from treated and untreated mice to show change in tumorsize (D) & (E) Micrograph of representative H-E stained sections of tumortissue. (F) Change in tumor weight. (G) TumorcellCount. The data represented as mean+S.D. for the three different experiments performed in triplicate. Significant difference ($p<0.05$) is indicated with the following symbols“*”when compared with Melanoma control.

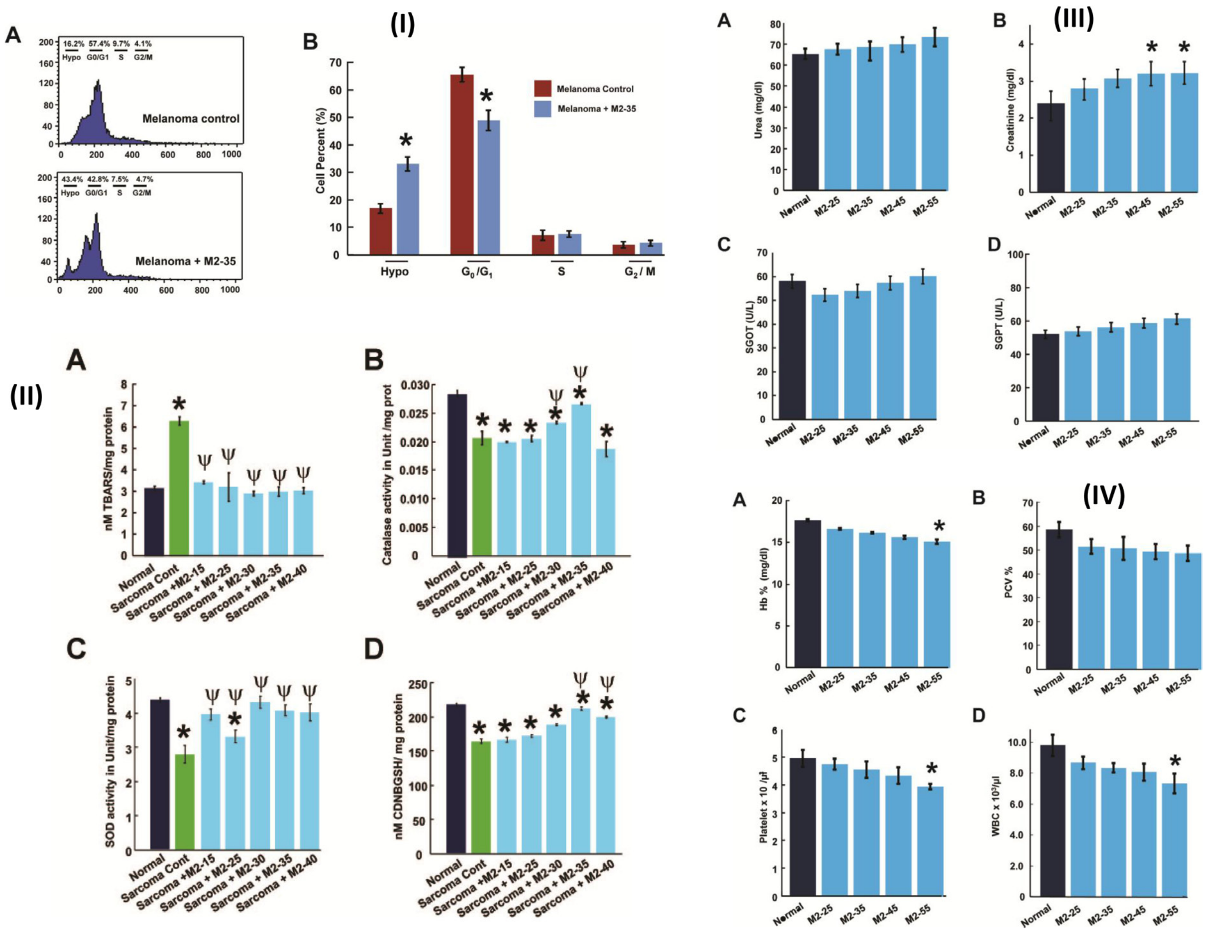

Supplementary Figure 2: (I) B-16 Melanoma tumor cell cycle phase distribution detected in a flowcytometer. (A) Histogram display of DNAcontent (x-axis, PI-fluorescence) vs. counts(y-axis) has been shown. (B) Bar diagram representation of cell cycle phase distribution of S-180 from different experimental groups. The data represented as mean+S.D. for the three different experiments performed in triplicate. Significant difference ($p<0.05$) is indicated with the following symbols"*"when compared with Melanoma control. Effect of various concentrations of M2 on hepatic oxidative stress/anti-oxidant enzymes. **(II)** (A) The levels of hepatic lipid peroxidation (B) CAT activity as assessed by measuring the breakdown of H_2O_2 spectrophotometrically at 240nm (C)SOD activity as measured based on pyrogallol auto oxidation inhibition and expressed as unit/mg of protein (D) Hepatic GST activity as measured by determining the increase in absorbance at 340nm with 1-chloro-2,4-dinitrobenzene (CDNB) as the substrate. Data represented in bar diagrams of mean $\pm$ SD of three independent experiments;Significant difference ($p<0.05$) is indicated with the following symbols"*"when compared with sarcoma control. Effect of M2 on serum biochemical markers of toxicity in tumor bearing mice. **(III)** (A) ALP level B) SGPT level C) SGOT level D) Creatinine level. Significant difference ($p<0.05$) is indicated with the following symbols "*"when compared with Normal, "φ"when compared with Sarcoma control. Effect of M2 on serum biochemical markers of toxicity in normal miceA) Urea level B) Creatinine level C) SGOT level D) SGPT level.

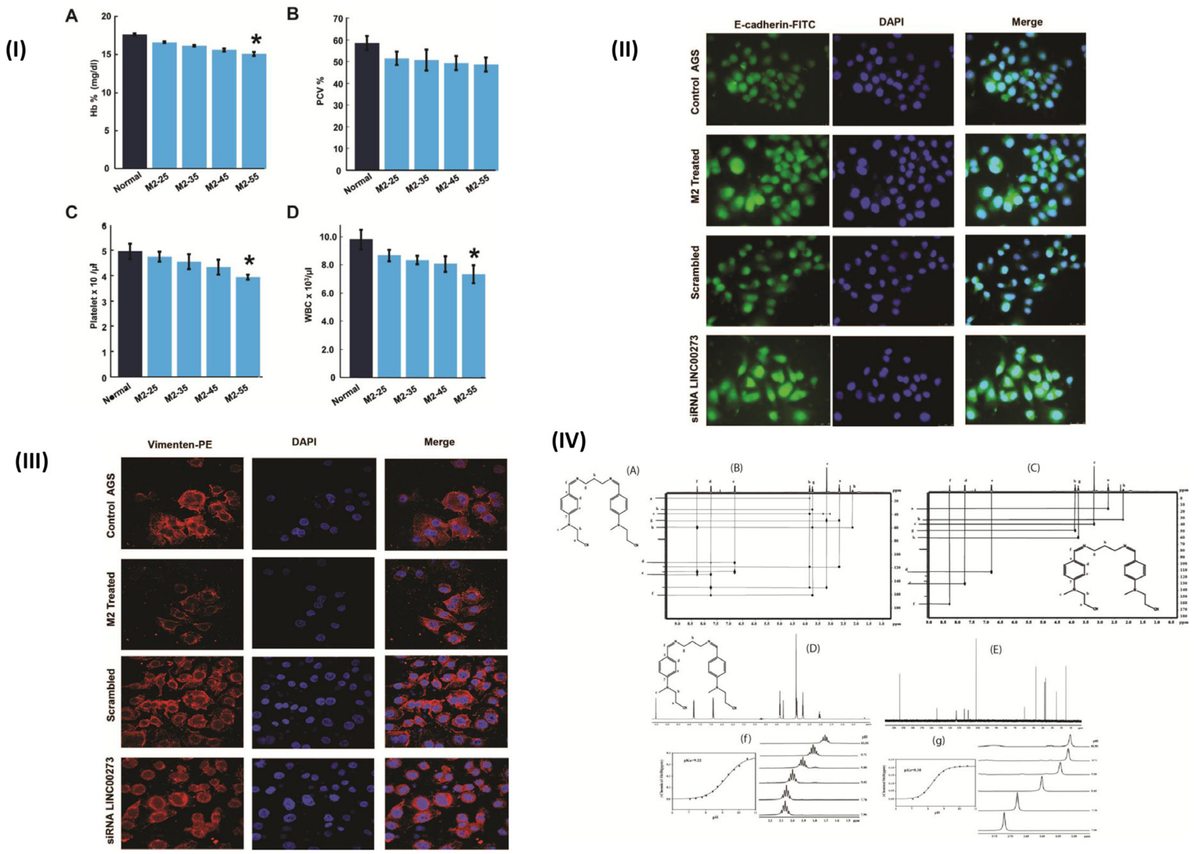

Supplementary Figure 3: **(I)** Effect of M2 on haematological parameters in normal mice (A) Hemoglobin percent (B) Percent Packed CellVolume (C) Platelet count (D) WBC count. **(II)** Expression of E-Cadherin (FITC labelled) (EMT marker) in AGS Cancer Cell line upon no treatment, M2, Si-RNA (scrambled), siRNA (LINC00273) treatment observed by Confocal microscopy. **(III)** Expression of E-Vimentin (PE labelled) (EMT marker) in AGS Cancer Cell line upon no treatment, M2, Si-RNA (scrambled), siRNA (LINC00273) treatment observed by Confocal microscopy. **(IV)** (A) 2D HMBC NMR spectra of M2. (B)2D HSQC NMR spectra of M2. (C) 1 D proton NMR spectra of M2. (D) 1D 13 C spectra NMR spectra of M2. (E) & (F) pKa profile of imine Nitrogen.

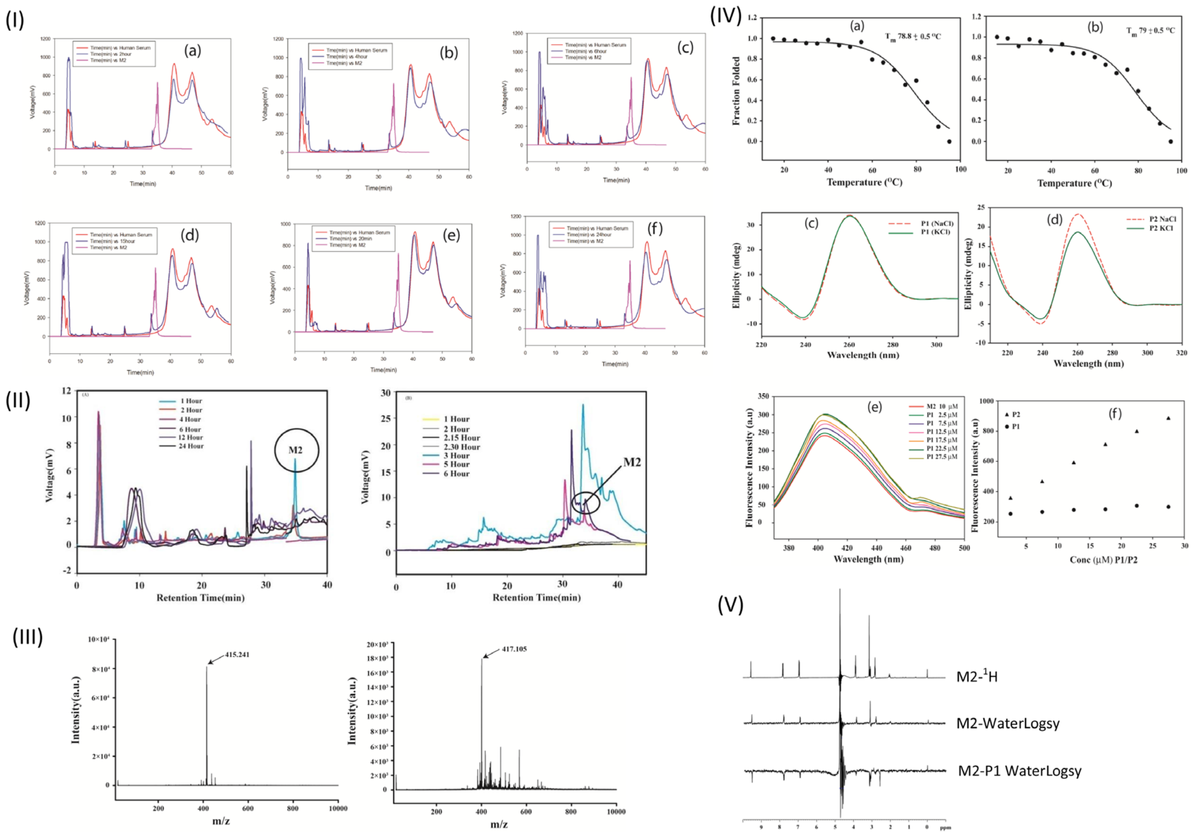

Supplementary Figure 4: (I) HPLC profile of human serum (treated with M2) collected at different time intervel. **(II)** Representative HPLCchromatogram of urineof M2 treated mice at different time interval (left panel). Representative HPLC chromatogram of urine of M2 treated mice at differential at different time interval (right panel). **(III)** MALDI spectra of pure M2 (left panel). MALDI spectra of M2 collected from M2 treated mice serum. **(IV)** (A) Melting curve of P1 (B) Melting curve of P1-M2 complex (1:3) (C) CD spectra of P1 in 100 mM KCl and 100 mM NaCl (D) CD spectra of P2 in 100 mM KCl and 100 mM NaCl (E) Fluorescence spectra of M2 titrated with increasing concentration of P1 (F) Comparison of Fluorescence intensity of M2 with increasing concentration of P1/P2. **(V)** (A) Reference 1D proton NMR spectra of M2 (top), WaterLOGSY 1D proton spectra of M2(middle), WaterLOGSY 1D proton spectra of M2-P1 complex (bottom).

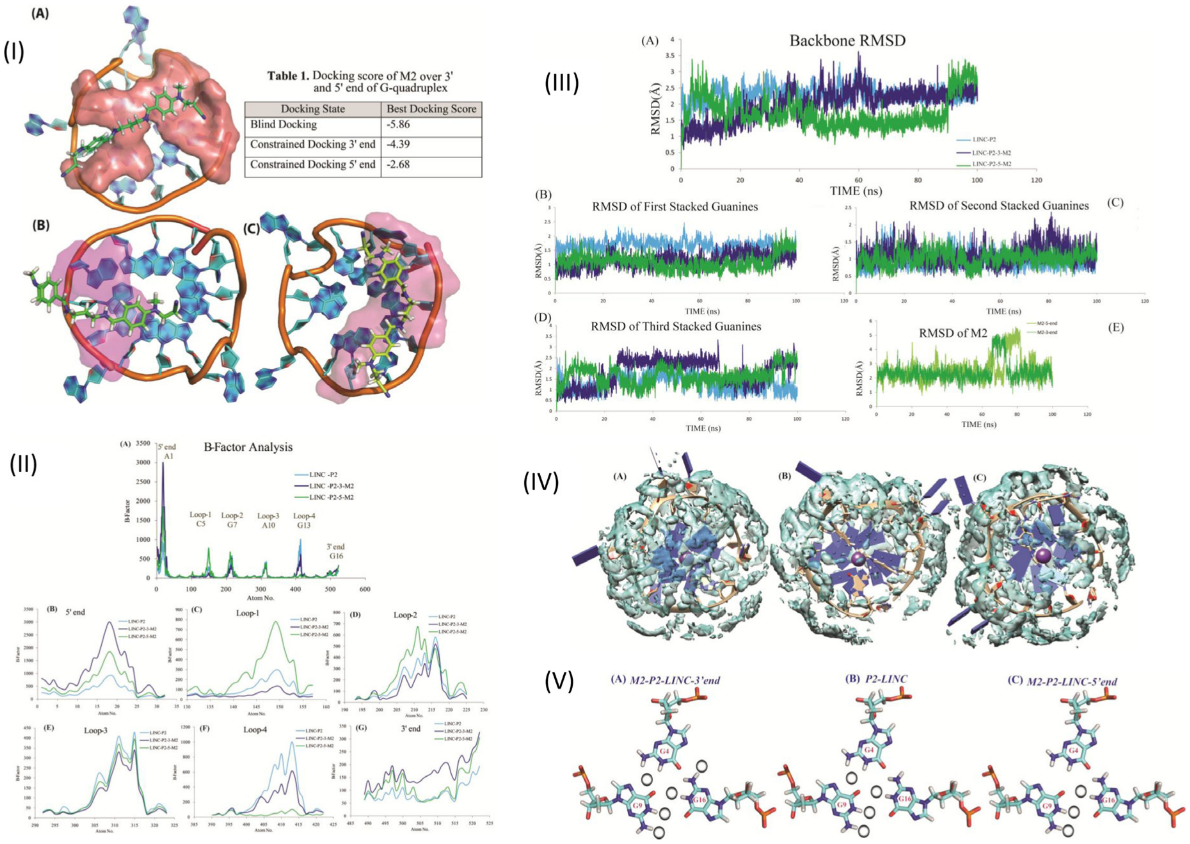

Supplementary Figure 5: **(I)** Docking analysis, (A) blind docking of M2 over P2-LINC where major binding is found at 3' end, (B) constraineddocking of M2 over 3' end, (C) constrained docking of M2 over 5' end, along with the respective docking scores enlisted in Table 1. **(II)** RMSD (Å) estimation per unit time (ns), for various fragments of all simulated systems (unbound state in sky blue color, M2-bound at 5' end in green color, M2-bound at 3' end in dark blue color); (A) backbone RMSD of overall structures (B) all atom RMSD of guanines involved in first G-stacking (C) all atom RMSD of guanines involved in second G-stacking (D) all atom RMSD of guanines involved in third G-stacking (E) RMSD of M2 when bound at 3'(green color) and 5'(lemon color) end regions. **(III)** B-Factor estimation at atomic level, (A) B-factor of entire sequence of P2-LINC in different complexation state, B-factor estimation for different fragments are elaborated further (B) B-factor of 5' end overhanging base A1, B-factor estimation for (C) Loop-1, (D) Loop-2, (E) Loop-3, (F) Loop-4 and (G) B-factor of 3' end base. **(IV)** Water grid density map of P2-LINC in different complexation states: (A) unbound P2-LINC, (B) M2-P2-LINC complex M2 bound at 3' end, (C) M2-P2-LINC complex M2 bound at 5' end. **(V)** Schematic representation of localization of the water molecules around the guanine triad (at the 3' end of P2-LINC) based on the water density map analysis.

(A)

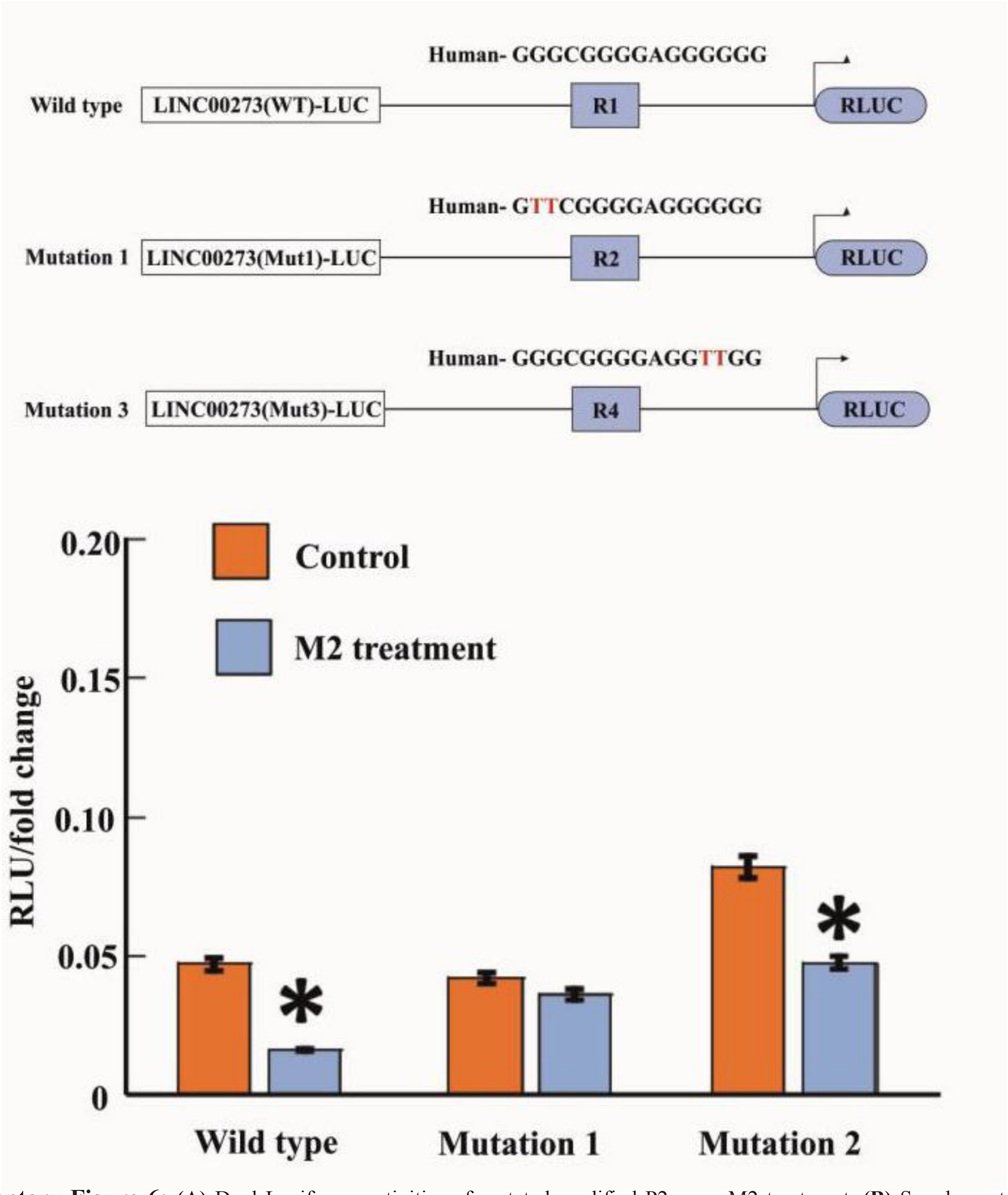

Supplementary Figure 6: (A) Dual Luciferase activities of mutated modified P2 upon M2 treatment. **(B)** Supplementary Table 1. MMPB(GB)SAcalculations of binding free energy components of M2 when bound to P2-LINC 5' and 3' end, analyzed over last 2ns simulation period.

Supplementary Table 1: MMPB(GB)SA calculations of binding free energy components of M2 when bound to P2-LINC 5' and 3' end, analyzed over last 2ns simulation period

*Energy (kcal/mol)	5'-P2-LINC	3'-P2-LINC
^{E}VDWAALS	-37.35±3.07	-28.70±3.74
^{E}EEL	-740.34±16.56	-717.36±22.75
^{E}PB	760.12±16.99	725.79±23.85
^{E}GB	749.11±16.69	615.33±14.84
^{E}NPOLAR	-2.81±0.22	-2.81± 0.23
^{E}SURF	-3.41±0.28	-2.88± 0.36
^{E}DISPER	0.00	0.00
$^{\Delta G}$Gas	-777.69±18.43	-746.07±24.28
$^{\Delta G}$SolvPB	757.01±16.92	722.97±23.71
$^{\Delta G}$SolvGB	745.70±16.57	726.21 ± 23.36
$^{\Delta G}$Binding EnergyPB	**-20.68±4.12**	**-23.09±2.21**
$^{\Delta G}$Binding EnergyGB	**-31.99±3.12**	**-19.86± 2.74**

$E_{VDWAALS}$: Non-Bonded van der Waals Energy
E_{EEL}: Non-Bonded Electrostatic Energy
E_{PB}: Polar solvation energy(PB)
$E_{GB:}$ Polar solvation energy(GB)
E_{NPOLAR}: Non polar solvation energy from repulsive solute-solvent interactions (PB)
E_{SURF}: Non polar solvation energy(GB)
E_{DISPER}: Non-polar contribution to solvation energy from attractive solute-solventinteractions(PB)
ΔG_{Gas}: $E_{VDWAALS}$ + E_{EEL} + Internal Energy
$\Delta G_{Solv:}$ Polar solvation energy + Non polar solvation energy
$\Delta G_{Binding\ Energy}$: Binding Free Energy($\Delta G_{Gas}$ + ΔG_{Solv})
The energies given are approximate values as predicted by the software and not measured experimentally.

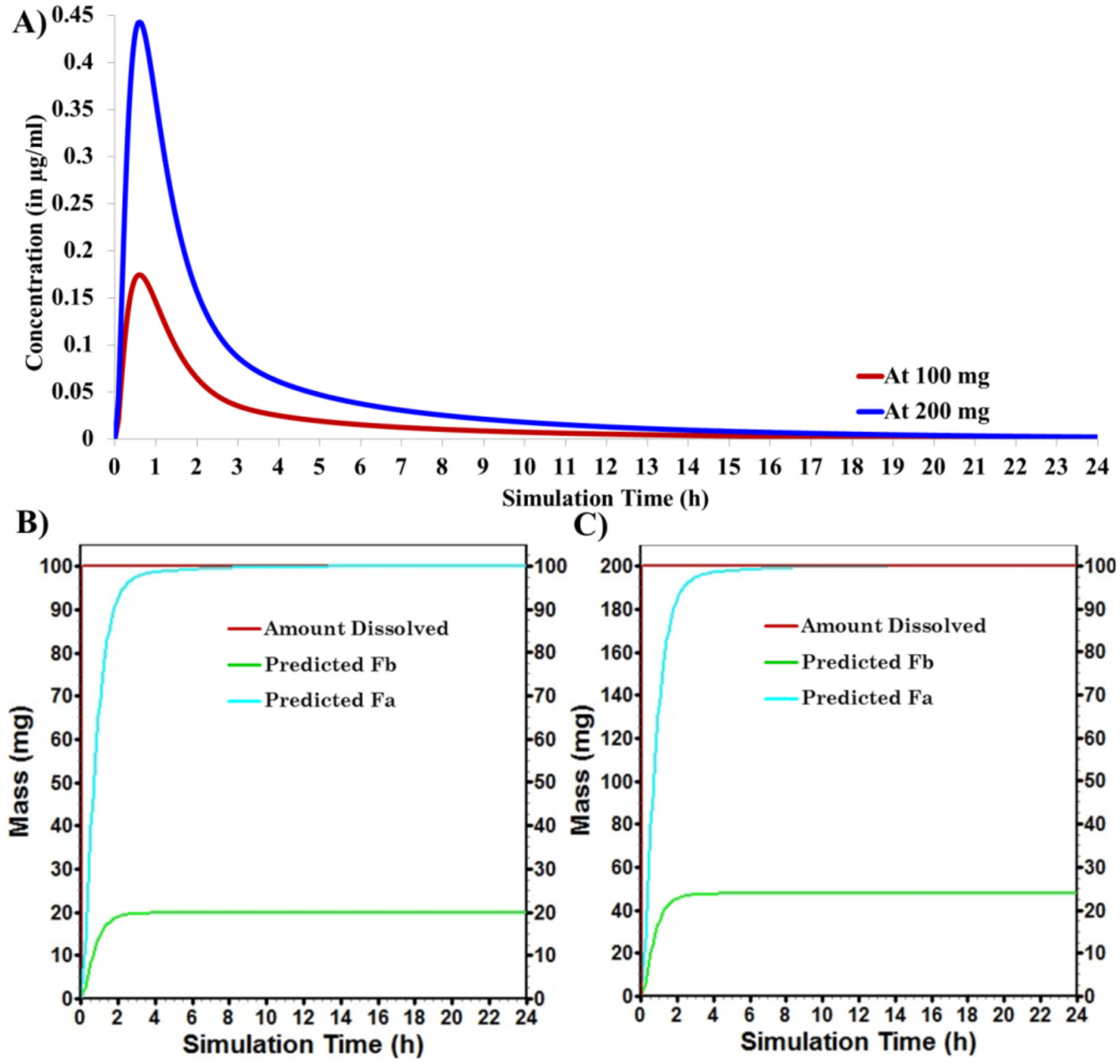

Figure pS1: Drug absorption analysis for M2 over the 24 h simulation. **(A)** C_{max} *vs.*time ; **(B)** Amount ofdrug absorbed *vs.* time at 100 mg concentration of M2 and; (B) Amount of drug absorbed *vs.* time at 200 mg concentration of M2. Fb : %bioavailability and Fa: Fraction absorbed.

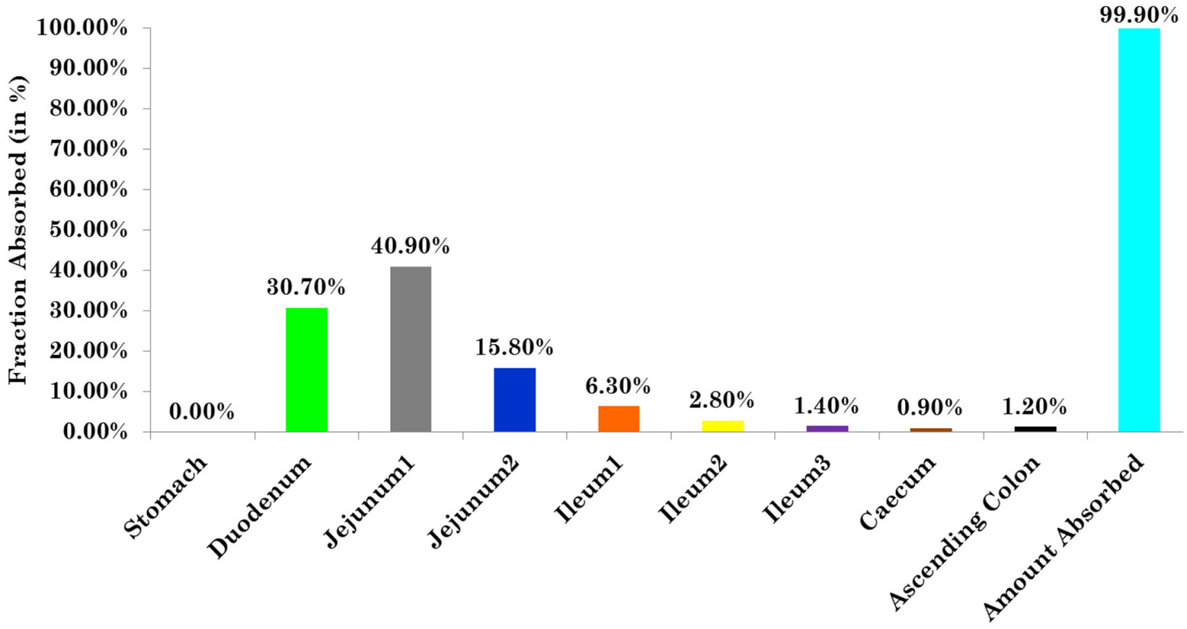

Figure pS2: Regional absorption of drug in different compartment during simulation of 24 h.

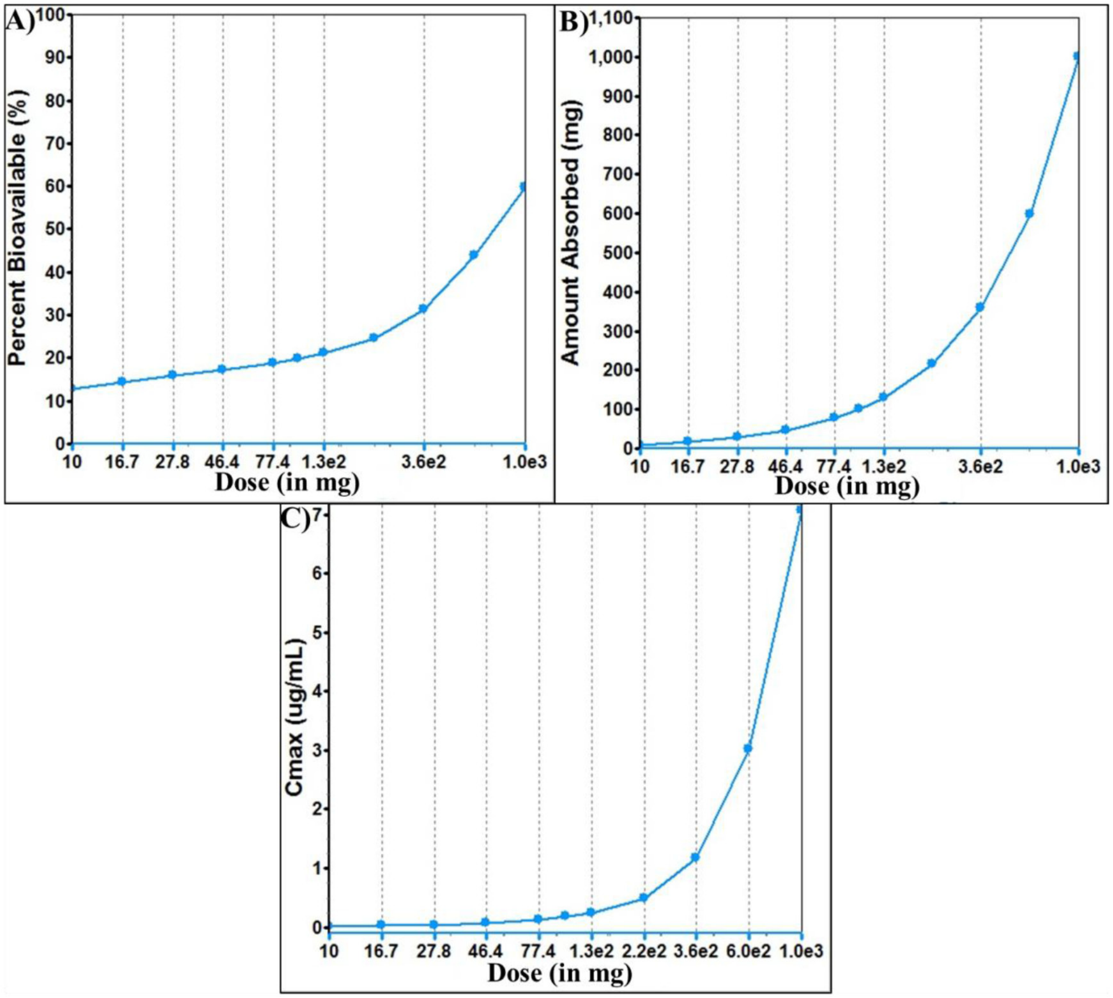

Figure pS3: Parameter sensitivity analysis (PSA) of (A) % bioavailability *vs.* dose of the drug; **(B)** amountabsorbed *vs.* dose of the drug and; **(C)** C_{max} *vs.* dose of the drug.

Table pS1: Predicted compound specific parameter estimates for M2

**Parameters**	***In silico* predicted**
Molecular Weight	426.65
LogP	3.70
Solubility at pH 9.31 (mg/mL)	0.24
Permeability (human P_{eff})	2.44
pK_a	8.82, 7.50, 6.52, 4.75
CL_{sys}(L/h)	7.22
Cblood/Cplasma	0.53
V_{ss} (L)	148.56
Particle density (g/ml)	1.20
Mean Precipitation Time (in sec)	900
Effective Particle Radius (in micron)	25.00

Table pS2: Parameter Output from GastroPlus™for M2

Parameter	Predicted Value
**At 100 mg dose**	
Absorption	99.87%
Bioavailability	19.89%
Cmax	0.17 µg/mL
Tmax	0.64 h
**At 200 mg dose**	
Absorption	99.87%
Bioavailability	23.92%
Cmax	0.44 µg/mL
Tmax	0.64 h
